# Early discontinuation vs. continuation of empirical antibiotics in patients with cancer and febrile neutropenia: a systematic review and meta-analysis of randomized controlled trials

**DOI:** 10.3389/fmicb.2026.1925996

**Published:** 2026-09-25

**Authors:** Juan Wang, Yuyi Tang, Ziquan Xia, Ruixuan Ye, Nawsherwan, Jie Hu

**Affiliations:** 1 School of Public Health and Health Sciences, Hubei University of Science and Technology, Wuhan, Hubei, China; 2 The First School of Clinical Medicine, Nanjing Medical University, Nanjing, China; 3 School of Clinical Medicine, Guilin Medical University, Guilin, China; 4 Department of Clinical Medicine, Kangda College of Nanjing Medical University, Lianyungang, Jiangsu, China; 5 School of Nursing, Shandong Xiehe University, Jinan, Shandong, China; 6 Cancer Center, Union Hospital, Tongji Medical College, Huazhong University of Science and Technology, Wuhan, Hubei, China

**Keywords:** antimicrobial stewardship, cancer, early antibiotic discontinuation, febrile neutropenia, systematic review and meta-analysis

## Abstract

**Background:**

Empirical broad-spectrum antibiotics are standard care for febrile neutropenia, but prolonged treatment until neutrophil recovery may increase antimicrobial resistance, toxicity, *Clostridioides difficile* infection, fungal superinfection, and healthcare burden. Whether antibiotics can be safely discontinued before neutrophil recovery remains uncertain.

**Methods:**

We systematically reviewed randomized controlled trials comparing early or short-course discontinuation of empirical antibacterial therapy with continuation until neutrophil recovery or an extended fixed course in patients with cancer and febrile neutropenia. MEDLINE, Embase, Cochrane CENTRAL, and ClinicalTrials.gov were searched from inception. Data were pooled using random-effects models. Risk of bias was assessed using the Cochrane RoB 2 tool. Co-primary outcomes were days of antibiotic therapy and all-cause mortality. The review was registered in PROSPERO (CRD420261434782).

**Results:**

Fifteen trials involving 1,692 participants (1,679 contributing to the mortality analysis) were included. Early discontinuation reduced antibiotic exposure by a mean of 3.43 days (95% CI − 5.59 to −1.27; I^2^ = 87.1%), with a larger reduction in children (−5.04 days, 95% CI − 8.55 to −1.54) than adults (−1.52 days, 95% CI − 3.49 to 0.44; subgroup *p* = 0.0002). All-cause mortality did not differ between groups (Peto odds ratio 1.23, 95% CI 0.64 to 2.36), and the absolute risk difference across all trials was null (0.00, 95% CI − 0.01 to 0.01; I^2^ = 0%). No significant differences were observed in new bloodstream infections, treatment failures, or fever recurrences.

**Conclusion:**

In clinically stable, afebrile patients with cancer-related febrile neutropenia, early antibiotic discontinuation shortened antibiotic exposure, particularly in children, without a detectable increase in mortality or major infectious complications. Larger adult trials are needed to confirm safety.

**Systematic review registration:**

https://www.crd.york.ac.uk/PROSPERO/view/CRD420261434782, CRD420261434782.

## Introduction

Febrile neutropenia is among the most common and potentially life-threatening complications of cancer treatment and remains a leading cause of unplanned hospitalization, broad-spectrum antibiotic exposure, and treatment-related morbidity in patients receiving cytotoxic chemotherapy, hematopoietic stem-cell transplantation (HSCT), or chimeric antigen receptor (CAR) T-cell therapy (Boccia et al., 2022). Because bacterial infection can progress rapidly when host defenses are impaired, prompt empirical antibacterial therapy is the cornerstone of management and is particularly critical in patients with hematological malignancies, profound or prolonged neutropenia, or clinical instability (Keck et al., 2022). Current guidance, therefore, continues to emphasize immediate initiation of empirical antibiotics in febrile neutropenic patients, while increasingly acknowledging that the appropriate duration of therapy once a patient is afebrile, clinically stable, and without a documented infection is far less certain (Keck et al., 2022; Lehrnbecher et al., 2023).

Traditionally, empirical antibiotics have been continued until neutrophil recovery, usually defined as an absolute neutrophil count above 500 cells/μL. This conservative strategy was intended to protect against occult or breakthrough infection during persistent neutropenia, but it prolongs exposure to broad-spectrum agents in a large proportion of episodes, often 30–50%, in which no infectious source is ever identified (Keck et al., 2022). Prolonged and cumulative antibiotic exposure is an important driver of antimicrobial resistance, which is rising among Gram-negative bloodstream pathogens in hematology and transplant populations and is associated with substantially higher mortality when empirical cover proves inadequate (Baccelli et al., 2025; Park et al., 2024). Beyond resistance, extended therapy contributes to *Clostridioides difficile* infection, disruption of the gut microbiome, drug toxicity, prolonged hospitalization, and increased cost—harms that accumulate across the repeated febrile episodes typical of these patients (Park et al., 2024).

Against this background, the optimal time to stop empirical antibiotics has become one of the central stewardship questions in supportive cancer care, and international recommendations diverge. The 2023 update of the international pediatric fever and neutropenia guideline introduced a conditional recommendation permitting discontinuation of empirical antibacterial therapy in clinically well, afebrile children with low-risk febrile neutropenia and negative blood cultures at 48 h, even without marrow recovery (Lehrnbecher et al., 2023). In adults, the 2024 AGIHO guideline likewise moved away from mandating treatment until neutrophil recovery, instead supporting early de-escalation or discontinuation after several days of defervescence and clinical stability, irrespective of the neutrophil count (Sandherr et al., 2025). Nonetheless, caution persists particularly for high-risk adults and for patients with persistent fever reflecting residual uncertainty about safety (Sandherr et al., 2025).

Several contemporary randomized controlled trials have strengthened the evidence base. In adults with hematological malignancy, the trial by de Jonge and colleagues (NCT02149329) showed that discontinuing carbapenem therapy after 3 days in fever of unknown origin was non-inferior to extended treatment for treatment failure while reducing antibiotic exposure (de Jonge et al., 2022). A randomized trial in recipients of HSCT or CAR T-cell therapy found that early de-escalation and discontinuation increased antibiotic-free days without compromising clinical outcomes (Ram et al., 2023). In children, randomized trials in low-risk disease (the DALFEN and ProFenC studies) and, more recently, in high-risk acute leukemia have indicated that antibiotics can be stopped earlier in carefully selected, clinically stable patients (Kumar et al., 2021; Srinivasan et al., 2024; Chellapuram et al., 2025). Evidence from related cohort, pilot, and step-down studies points in the same direction, including a retrospective cohort that stopped antibiotics after 72 h in acute myeloid leukemia (Schauwvlieghe et al., 2021), an antimicrobial de-escalation-and-discontinuation program in high-risk patients (la Martire et al., 2018), a procalcitonin-guided pediatric pilot (Morgan and Phillips, 2022), and a first-day oral step-down trial in low-risk children (Brack et al., 2012). However, individual trials remain underpowered for the rare but decision-critical safety outcomes all-cause mortality, bloodstream infection, intensive-care admission, and invasive fungal infection, and previous syntheses, including the 2019 Cochrane review, have been constrained by few trials, heterogeneous populations, and predominantly low-certainty evidence (Stern et al., 2019; Ishikawa et al., 2023; Chen et al., 2025).

An updated systematic review and meta-analysis of randomized controlled trials is therefore warranted to consolidate the expanded evidence and to quantify both benefit and harm with greater precision. We evaluated whether early discontinuation of empirical antibacterial therapy before neutrophil recovery reduces antibiotic exposure without increasing mortality, treatment failure, bloodstream infection, or other infectious complications in patients with cancer and febrile neutropenia, and we appraised the certainty of this evidence across adult and pediatric populations.

## Method

### Protocol and registration

This systematic review and meta-analysis followed a prespecified protocol and is reported in accordance with the PRISMA 2020 statement (Page et al., 2021). The protocol was registered in PROSPERO (CRD420261434782). We compared early discontinuation of empirical antibacterial therapy before neutrophil recovery with continuation until neutrophil recovery or a longer fixed course in patients with cancer and febrile neutropenia.

### Eligibility criteria

We included randomized controlled trials enrolling adults or children with cancer, solid tumors, or hematological malignancies, including recipients of hematopoietic stem-cell transplantation or CAR T-cell/cellular therapy who developed febrile neutropenia, and comparing early or short-course discontinuation of empirical systemic antibacterial therapy with continuation until neutrophil recovery or an extended fixed duration. Febrile neutropenia was defined as in each trial, and both high- and low-risk episodes were eligible. Eligible discontinuation strategies included fixed short courses, cessation after defervescence and clinical stability, biomarker-guided stopping, and early de-escalation followed by discontinuation. We excluded non-randomized and observational studies, reviews, case reports or series, studies of non-cancer or afebrile neutropenia, antibacterial prophylaxis without fever, evaluations of antifungal or antiviral duration only, and comparisons of antibacterial spectrum that did not shorten treatment duration.

### Search strategy and study selection

We searched MEDLINE/PubMed, Embase, Cochrane CENTRAL, and the ClinicalTrials.gov trial register from inception to 1 June 2026, restricted to English-language reports. Search concepts combined febrile neutropenia, antibiotic discontinuation or treatment duration, and randomized trials; the full strategy for each source is provided in Supplementary Methods S1. Reference lists of eligible trials and relevant reviews were hand-searched. Titles, abstracts, and full texts were screened independently by two reviewers (Xia Ziquan and Ye Ruixuan), with disagreements resolved by discussion.

### Data extraction

Two reviewers independently extracted data using a structured form, capturing trial characteristics (name, year, country, setting, design), population (age group, cancer type, risk category), the intervention stopping rule and comparator, sample size, follow-up, and outcome data. Means, standard deviations, and analyzed sample sizes were extracted for continuous outcomes, and event counts with denominators for dichotomous outcomes. Where trials reported medians with ranges or interquartile ranges, means and standard deviations were estimated using established methods (Wan et al., 2014), and the influence of these reconstructions was examined in sensitivity analyses. Trial-level characteristics, including population and episode classification, the intervention and its stopping criteria, the randomized and analyzed populations, and the outcome definitions applied, are presented in Supplementary Tables S1–S6. For each outcome, we used the denominator of the population actually analyzed for that outcome, which in some trials is smaller than the number randomized; randomized and analyzed denominators for every trial and outcome are reconciled in Supplementary Table S4.

### Outcomes

The co-primary outcomes were antibiotic consumption and all-cause mortality. Antibiotic consumption was analyzed as total days of antibiotic therapy, with antibiotic-free days within a 28-day window analyzed where reported; all-cause mortality was assessed at the latest reported follow-up. Secondary outcomes were treatment failure (defined as in each trial), fever recurrence, new bloodstream infection, invasive fungal infection, multidrug-resistant organism acquisition, and hospital readmission. For bloodstream infection, the primary analysis was restricted to new, post-randomization bacteremia in trials enrolling patients afebrile at randomization.

### Risk of bias and certainty of evidence

Two reviewers independently assessed risk of bias with the Cochrane RoB 2 tool (Sterne et al., 2019), resolving disagreements by discussion. The certainty of evidence for the co-primary and key secondary outcomes was rated with the GRADE framework (Guyatt et al., 2008), considering risk of bias, inconsistency, indirectness, imprecision, and publication bias.

### Statistical analysis

All analyses were performed in R (version 4.3.1) using the *meta* and *dmetar* packages (Balduzzi et al., 2019; Harrer et al., 2021). In keeping with the registered protocol, outcomes reported by at least two trials were pooled using random-effects meta-analysis with the restricted maximum likelihood (REML) estimator for the between-study variance (τ^2^) and the Hartung–Knapp–Sidik–Jonkman (HKSJ) correction of confidence intervals, which accounts for uncertainty in τ^2^ and is particularly important when few trials are available; common-effect estimates are presented alongside for comparison. Dichotomous outcomes were expressed as risk ratios and continuous outcomes as mean differences, each with 95% confidence intervals. For rare-event outcomes (all-cause mortality and invasive fungal infection), for which the risk ratio is unstable, Peto odds ratios were used, and all-cause mortality was additionally summarized as an absolute risk difference across all included trials because several reported no deaths. Heterogeneity was quantified with the I^2^ statistic and τ^2^ and assessed with the Q-test (*p* < 0.10), and prediction intervals were reported where informative. A prespecified subgroup analysis by age group (adult vs. pediatric) was performed for antibiotic consumption. Sensitivity analyses comprised common-effect modeling, leave-one-out estimation, and restriction to trials reporting means directly (excluding trials in which means were reconstructed from medians). Small-study effects were examined using a contour-enhanced funnel plot for the only outcome with at least 10 contributing trials (treatment failure).

### Differences between the protocol and the review

The review followed the registered protocol (PROSPERO CRD420261434782) with the following changes, made in response to the available data. First, all-cause mortality had been registered with the risk ratio as the effect measure; because deaths were rare and several trials recorded none, we used the Peto odds ratio, which is better suited to sparse binary data, supplemented by an absolute risk difference. In sensitivity analyses, each dichotomous outcome was additionally pooled using Mantel–Haenszel methods without continuity correction (prespecified), and, *post hoc*, the historical trials that discontinued antibiotics during persistent fever (Björnsson et al., 1977; Pizzo et al., 1979; Pizzo et al., 1982) were analyzed separately from contemporary trials that stopped only after defervescence and clinical stability (Supplementary Table S7). Second, trial sequential analysis and Egger’s regression test, although prespecified, were not performed: most outcomes were informed by fewer than ten small trials, a setting in which the required-information-size and boundary estimates of trial sequential analysis, and regression-based tests of funnel-plot asymmetry, are unreliable and may mislead; small-study effects were instead assessed graphically for the only outcome with at least ten trials (treatment failure). A formal reporting bias assessment with the ROB-ME tool was not undertaken for the same reason. Third, several prespecified secondary outcomes (infection-related mortality, sepsis, intensive-care admission, *Clostridioides difficile* infection, and length of stay), the planned meta-regression, and subgroup analyses by risk stratum, episode type, decision rule, and comparator type could not be conducted because too few trials reported the necessary data; the prespecified age subgroup was retained. Finally, the executed search was broader than the sources listed at registration and included Embase.

## Results

### Study selection

The study-selection process is shown in the PRISMA 2020 flow diagram (Figure 1). The search identified 5,978 records (5,777 from databases MEDLINE/PubMed, *n* = 1,671; Embase, *n* = 3,499; Cochrane CENTRAL, *n* = 607; and 201 from ClinicalTrials.gov). After 5,515 records were removed before screening (5,081 duplicates, 289 flagged as ineligible by automation tools, and 145 removed for other reasons), 463 records were screened on title and abstract, and 71 were excluded. Of the 392 reports sought for retrieval, 42 could not be obtained, and 350 reports underwent full-text assessment, of which 335 were excluded, most often for wrong study design (*n* = 206), ineligible population (*n* = 83), ineligible comparator (*n* = 20), ineligible intervention (*n* = 18), or no outcome of interest (*n* = 8). Fifteen randomized controlled trials met the eligibility criteria.

**Figure 1:**
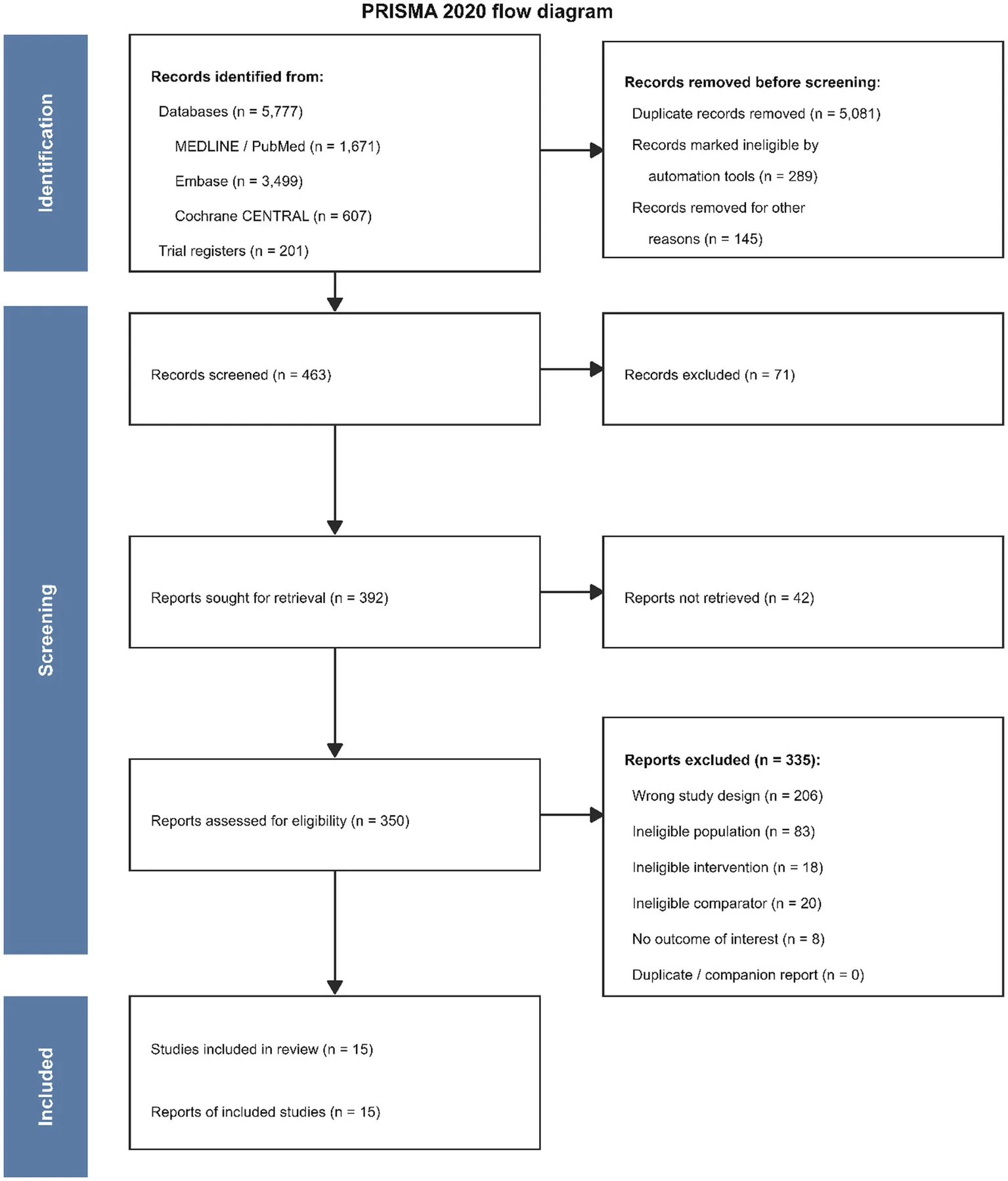
PRISMA 2020 flow diagram of study selection.

### Study characteristics

The characteristics of the 15 trials are summarized in Table 1. They were published between 1973 and 2026 and together randomized more than 1,600 patients; in the most inclusive analysis (all-cause mortality, all 15 trials), 832 participants/episodes were allocated to early discontinuation and 847 to continuation. Five trials enrolled adults, eight enrolled children, and two enrolled mixed-age populations, all with cancer, who developed neutropenia and fever. In the intervention arm, empirical antibacterials were stopped early or after a short, fixed course, most often once patients had been afebrile and clinically stable for 48–72 h, irrespective of neutrophil recovery (one trial used procalcitonin guidance), whereas the comparator arm continued antibiotics until neutrophil recovery or for a longer fixed course. Two trials reported outcomes per febrile episode (Klaassen et al., 2000; Vissing et al., 2026) and two enrolled children with documented viral respiratory infection (Santolaya et al., 2017; Torres et al., 2024). Because not every trial reported every outcome, the contributing trials and participants are provided separately for each analysis.

**Table 1:** Characteristics of the included randomized controlled trials (*n* = 15).

Trial	Country; setting	Design	Population (age; disease); risk	Intervention (early-discontinuation rule)	Comparator	N (I/C)
Adult trials						
Rodriguez et al. (1973)	USA; single	Open-label RCT	Adult (≥15 y); cancer; high	Stop on day 4 (fever of unknown origin)	Continue to day 14	81 (41/40)^7^
Björnsson et al. (1977)	USA; single	Open-label RCT	Adult; cancer; high	Stop for persistent fever (day 3)	Continue + chloramphenicol/clindamycin (10 d)	17 (6/11)
Aguilar-Guisado et al. (2017)	Spain; multicenter	Open-label RCT	Adult; hematological malignancy/HSCT; high	Stop after ≥72 h afebrile + stable, any ANC	Continue to ANC recovery	157 (78/79)
de Jonge et al. (2022)	Netherlands; multicenter	Open-label RCT, NI	Adult; hematological malignancy/HSCT; high	Short carbapenem; stop after defervescence + stable	Extended carbapenem to ANC recovery	292 (144/137)^1^
Ram et al. (2023)	Israel; single	Open-label RCT	Adult; post-HSCT / CAR T-cell; high	Early de-escalation + stop	Continue to recovery	110 (59/51)^8^
Pediatric trials						
Santolaya et al. (1997)	Chile; single	Open-label RCT	Pediatric; mixed cancer; low / mixed	Stop on day 3 (FUO, stable)	Continue to fever/neutropenia resolution	75 (36/39)
Klaassen et al. (2000)	Canada; single	Double-blind RCT	Pediatric; cancer (ALL predominant); low	Stop at discharge (placebo)	Oral antibiotics to ANC > 500 or 14 d	73 (36/37)^2^
Santolaya et al. (2017)	Chile; 5 centers	Open-label RCT	Pediatric; cancer + viral RTI; mixed	Stop at 48 h (virus+, stable)	Continue to episode end (7 d)	176 (84/92)
Kumar et al. (2021)	India; single	Open-label RCT	Pediatric; cancer; low	Stop after 24–48 h afebrile	Oral antibiotics to recovery	75 (38/37)
Srinivasan et al. (2024)	India; single	Open-label RCT	Pediatric; cancer; low	Procalcitonin-guided stop	Standard continuation	46 (23/23)
Torres et al. (2024)	Chile; 7 centers	Open-label RCT, NI	Pediatric; cancer + viral RTI; mixed	Stop at 48–72 h (virus+, stable)	Continue to episode end	139 (69/70)
Chellapuram et al. (2025)	India; single	Open-label RCT	Pediatric (2–18 y); acute leukemia; high	Stop after ≥72 h afebrile + stable	Continue to ANC ≥ 500	280 (140/138)^3^
Vissing et al. (2026)	Denmark; multicenter	Open-label RCT	Pediatric; cancer (ALL/AML); high	Stop after 48 h defervescence, any ANC	Continue to ANC recovery (max 10 d)	88 (45/43)^4^
Mixed-age trials						
Pizzo et al. (1979)	USA; single	Open-label RCT^5^	Mixed (1–33 y); leukemia / solid tumor; high	Stop for persistent fever	Continue antibiotics	33 (17/16)
Pizzo et al. (1982)	USA; single	Open-label RCT	Mixed (2–30 y); cancer; high	Stop for persistent fever (day 7)	Continue ± amphotericin B	50 (16/34)^6^

I/C, intervention/control. N is the number randomized, with intervention/control denominators analyzed in parentheses.

^1^de Jonge et al. (2022): 292 randomized; 281 analyzed (144/137). ^2^Klaassen et al. (2000): 73 febrile episodes in 54 children. ^3^Chellapuram et al. (2025): 280 randomized; 278 analyzed. ^4^Vissing et al. (2026): 88 febrile episodes in 70 children. ^5^Pizzo et al. (1979): prospective study with a randomized subset. ^6^Pizzo et al. (1982): control arm is the combined extended-treatment groups. ^7^Rodriguez et al. (1973): 81 episodes of fever of unknown origin in 76 patients. The trial performed two separate day-4 randomizations — one among the 56 afebrile episodes (30 discontinue/26 continue) and one among the 25 episodes with persistent fever (11 discontinue/14 continue). Both strata are pooled in all analyses, giving 41 episodes allocated to discontinuation and 40 to continuation; these denominators are used for every outcome. ^8^Ram et al. (2023): 110 patients randomized before admission (59/51); 91 subsequently developed febrile neutropenia and received empirical antibacterial therapy (50/41). All outcomes use the intention-to-treat cohort (59/51) except days of antibiotic therapy, which is defined only in patients who received empirical therapy and therefore uses 50/41.

### Antibiotic consumption (co-primary outcome)

Six trials (480 patients assigned to early discontinuation and 461 to continuation) reported the days of antibiotic therapy (Figure 2). Early discontinuation reduced antibiotic exposure, with a random-effects mean difference of −3.43 days (95% CI − 5.59 to −1.27; common-effects model −2.86 days, 95% CI − 3.47 to −2.25). Between-study heterogeneity was high (I^2^ = 87.1%, τ^2^ = 3.57), and the 95% prediction interval (−8.76 to 1.90) crossed the null, so the age-stratified estimates are the more informative summary. The effect differed significantly by age (test for subgroup differences *p* = 0.0002): in adults, the reduction was modest, and its confidence interval included no difference (mean difference −1.52 days, 95% CI − 3.49 to 0.44; I^2^ = 8.1%), whereas in children it was large and significant (−5.04 days, 95% CI − 8.55 to −1.54; I^2^ = 69%).

**Figure 2:**
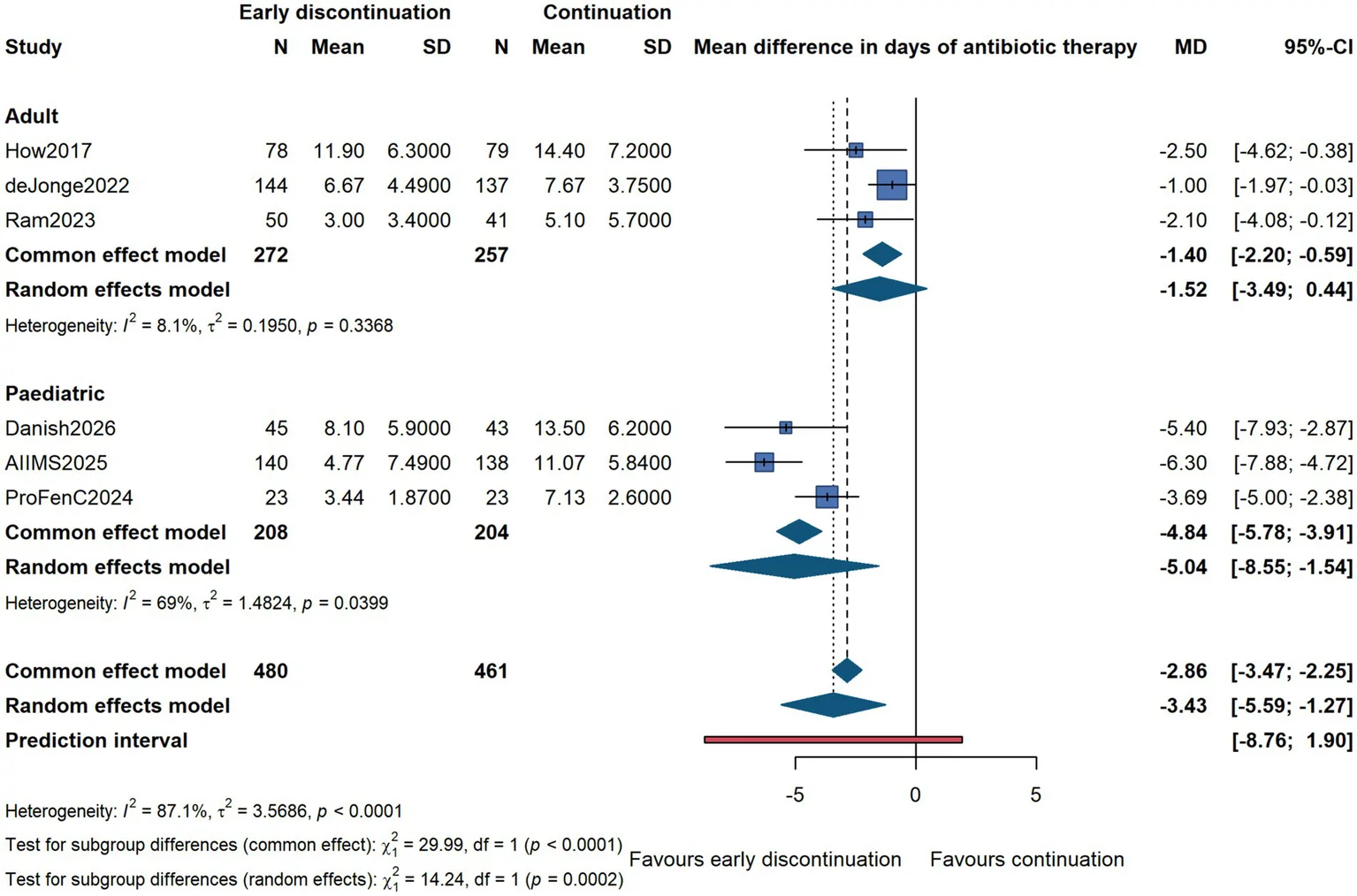
Days of antibiotic therapy: mean difference between early discontinuation and continuation, stratified by age; random-effects model. For Ram et al. (2023), the denominators are the per-protocol cohort (50 vs. 41), i.e., the patients who developed febrile neutropenia and received empirical antibacterial therapy, since the duration of therapy is undefined in the remaining randomized patients.

Two sensitivity analyses indicate that this heterogeneity is largely explained rather than residual (Supplementary Figure S1). Leave-one-out analysis produced mean differences from −2.76 to −4.01 days, all remaining significant; and restricting the analysis to the four trials that reported means directly left the estimate essentially unchanged (−3.34 days, 95% CI − 5.40 to −1.28) while reducing heterogeneity to I^2^ = 39.1%, showing that much of the dispersion arose from the two trials whose means were reconstructed from medians. Re-expressed as antibiotic-free days within a 28-day window (2 trials), the direction was concordant and again favored early discontinuation (common-effect mean difference +3.69 days, 95% CI 2.07 to 5.32).

### All-cause mortality (co-primary outcome)

Eight trials reporting at least one death (501 vs. 506 patients) were pooled using the Peto odds ratio (Figure 3). Mortality did not differ between groups (odds ratio 1.23, 95% CI 0.64 to 2.36; common-effect 1.15, 95% CI 0.66 to 1.99; I^2^ = 21.5%; prediction interval 0.35 to 4.38). Because deaths were rare and seven trials reported none, the analysis was repeated as a risk difference across all 15 trials (832 vs. 847 patients/episodes), which gave a precise estimate centered on the null (0.00, 95% CI − 0.01 to 0.01; I^2^ = 0%; study-level data in Supplementary Table S1). The narrow absolute interval (approximately ±1 percentage point) makes a clinically significant increase in mortality with early discontinuation unlikely, although the trials were individually underpowered for this outcome.

**Figure 3:**
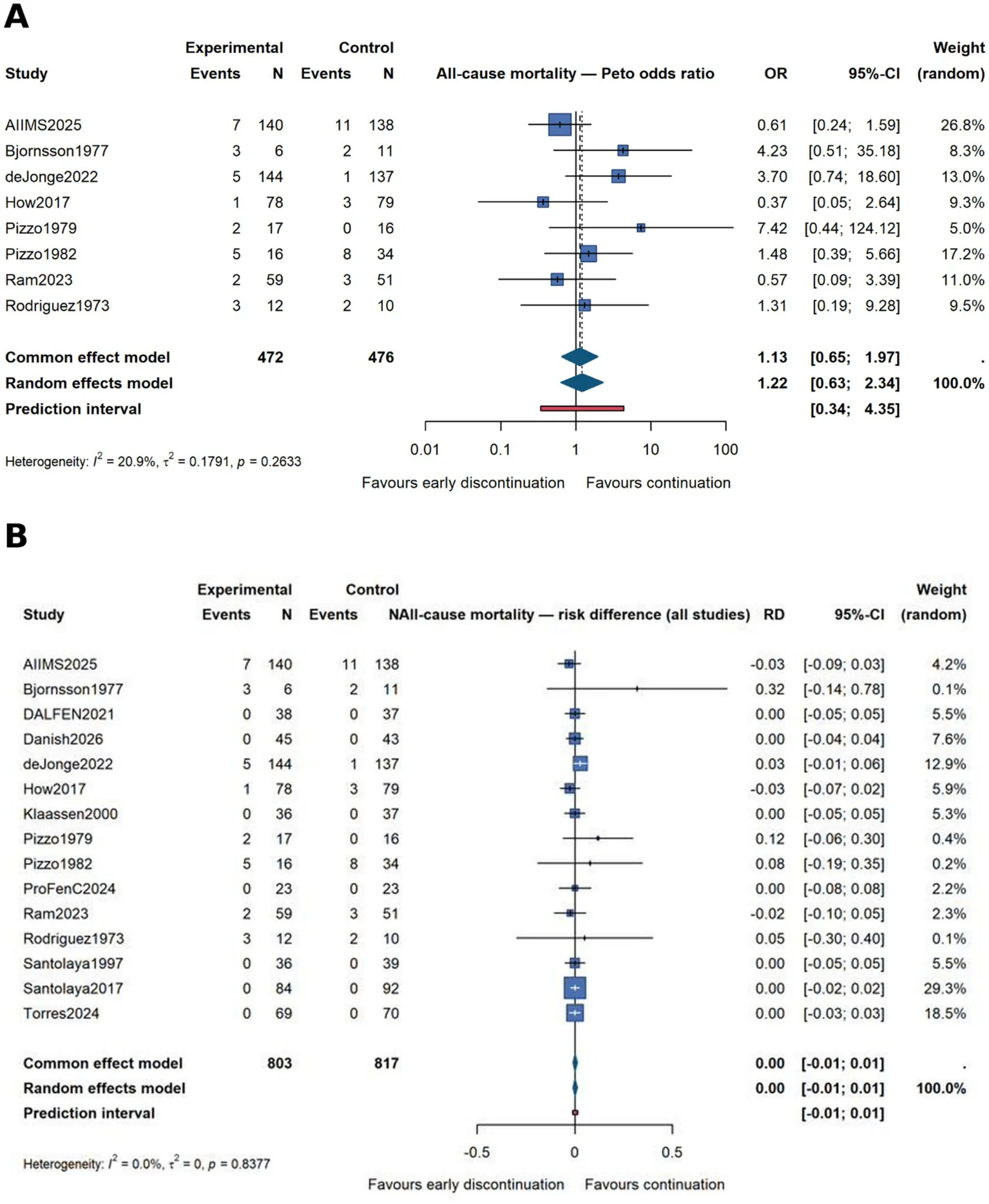
All-cause mortality. **(A)** Peto odds ratio (early discontinuation vs. continuation), pooled across the eight trials reporting at least one death. **(B)** Risk difference across all 15 trials (832 vs. 847 participants/episodes), random-effects (REML) model with double-zero trials retained; events were counted at each trial’s latest reported follow-up. OR, odds ratio; RD, risk difference; CI, confidence interval. For Rodriguez 1973, the denominators are the full randomized groups (41 vs. 40 episodes), pooling the trial’s two day-4 randomization strata (afebrile and persistent fever).

### Risk of bias

Two reviewers independently assessed risk of bias for the key outcomes using the Cochrane RoB 2 tool, resolving disagreements by discussion; the per-study judgments are shown in the risk-of-bias traffic-light figure (Supplementary Figure S2), with the complete domain-level judgments and supporting rationale in Supplementary Table S8. The contemporary trials were generally at low risk of bias or raised some concerns, the latter chiefly from the open-label design affecting antibiotic consumption and other subjectively influenced outcomes. The five oldest trials (Pizzo et al., 1979; Pizzo et al., 1982; Rodriguez et al., 1973; Santolaya et al., 1997; Björnsson et al., 1977) were at high risk, owing to an unclear randomization process and deviations from intended interventions; missing outcome data were almost uniformly low risk.

### Bloodstream infection

The bloodstream-infection definition was harmonized before pooling: the primary analysis was restricted to trials that enrolled patients afebrile at randomization and counted new, post-randomization bacteremia, the scenario relevant to current practice. In that analysis (3 trials, 362 vs. 354 patients), there was no difference between groups (risk ratio 0.97, 95% CI 0.34 to 2.77; common-effect 0.98, 95% CI 0.61 to 1.57; I^2^ = 0.3%; Figure 4); the wide random-effects interval reflects the small number of events and does not exclude a clinically meaningful difference in either direction. A pre-specified sensitivity analysis that added older trials stopping antibiotics during persistent fever (6 trials) shifted the estimate upward but remained non-significant and more heterogeneous (risk ratio 1.59, 95% CI 0.55 to 4.63; I^2^ = 45.8%; Supplementary Table S1), consistent with occult-infection unmasking in that obsolete strategy rather than a hazard of contemporary early discontinuation.

**Figure 4:**
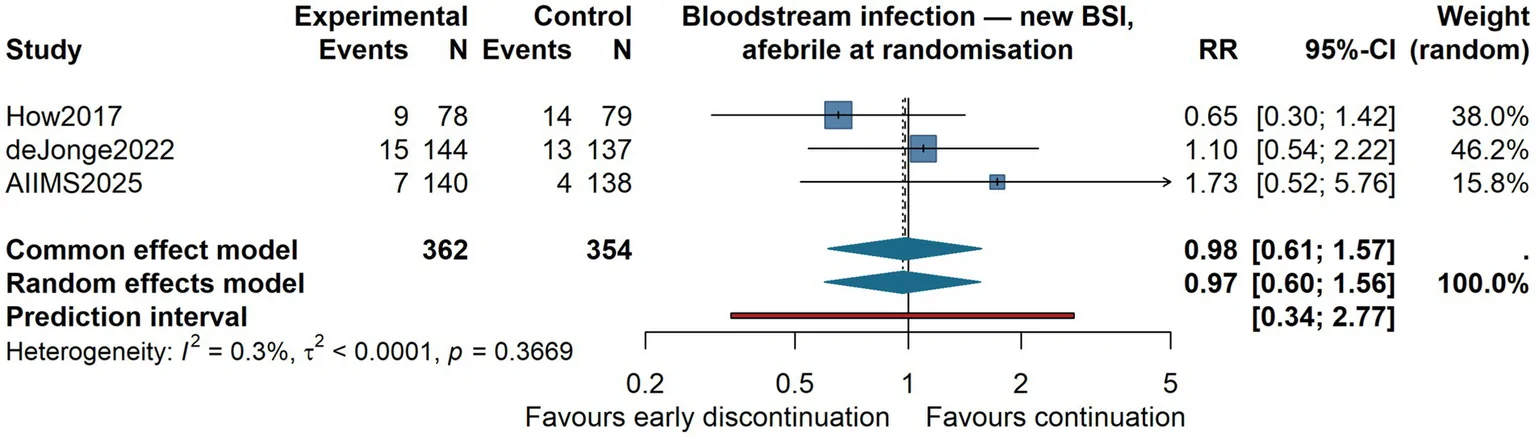
New bloodstream infection in trials enrolling patients afebrile at randomization: risk ratio.

### Treatment failure

Twelve trials (641 vs. 655 patients) reported treatment failure (Figure 5). The pooled estimate did not differ between groups (risk ratio 1.13, 95% CI 0.80 to 1.61; common-effect 1.13, 95% CI 0.86 to 1.48; I^2^ = 11.9%; prediction interval 0.62 to 2.07), although the confidence interval is compatible with a modest increase. A contour-enhanced funnel plot, produced for treatment failure as the only outcome with at least 10 trials, showed no marked asymmetry (Supplementary Figure S3).

**Figure 5:**
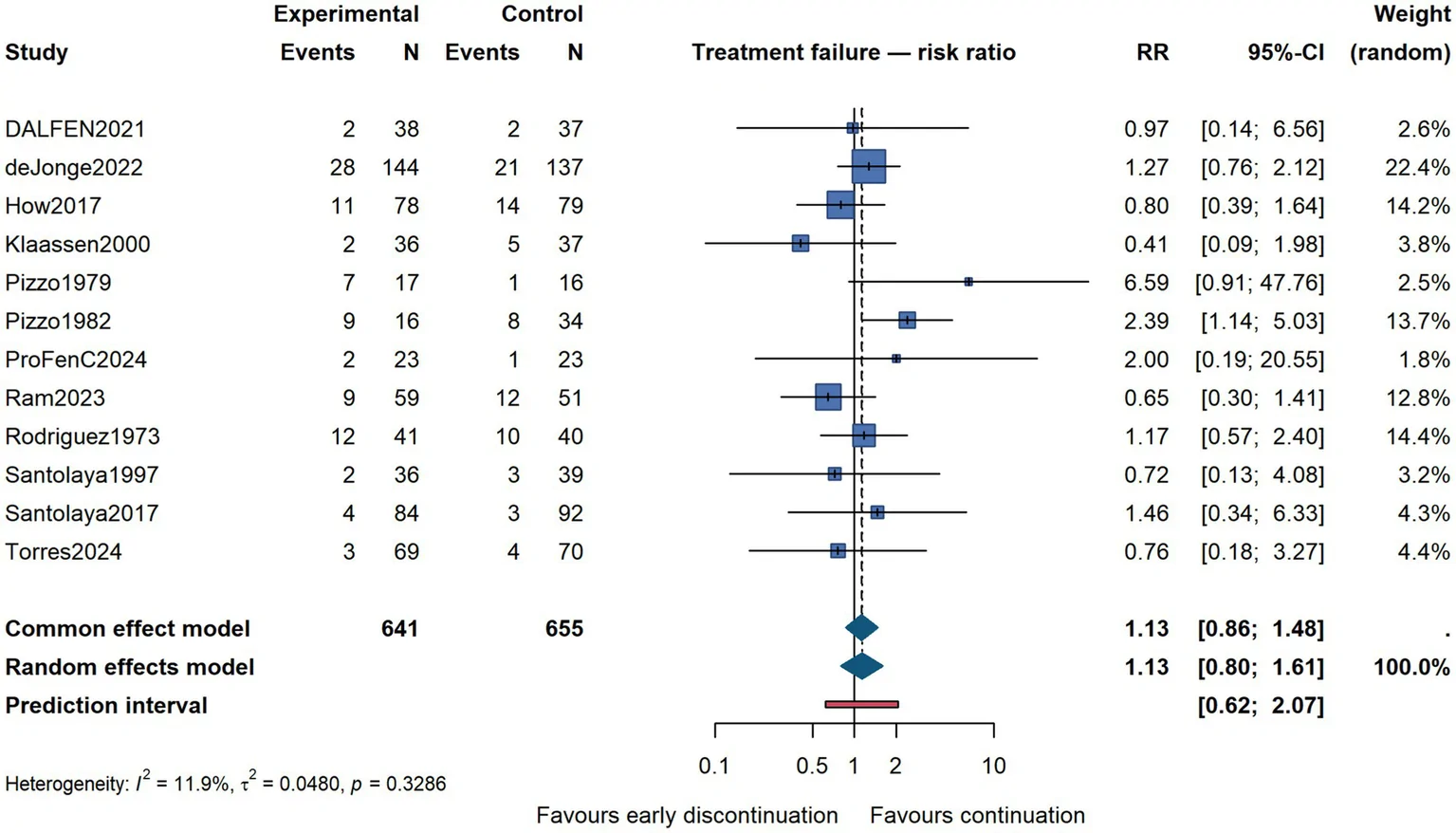
Treatment failure: risk ratio (early discontinuation vs. continuation).

### Fever recurrence

Nine trials (671 vs. 664 patients) reported fever recurrence, with no difference between groups and low statistical heterogeneity (risk ratio 1.08, 95% CI 0.88 to 1.33; common-effect 1.09, 95% CI 0.90 to 1.34; I^2^ = 0%; prediction interval 0.86 to 1.36); the relatively narrow interval makes a large increase in recurrent fever unlikely. Study-level data are given in Supplementary Table S1.

### Invasive fungal infection

Six trials (302 vs. 317 patients) reported invasive fungal infection (Peto odds ratio). The summary estimate was not significant (1.56, 95% CI 0.42 to 5.83; common-effect 1.04, 95% CI 0.54 to 1.98), but heterogeneity was substantial (I^2^ = 67.9%) and driven by one small, high-risk trial (Björnsson et al., 1977, odds ratio 32.69), with the remaining trials distributed around the null. Given the sparse events, the heterogeneity, and the clinical importance of this outcome in neutropenia, the evidence is insufficient to confirm or exclude an effect, and this result should be interpreted cautiously (Supplementary Table S1).

### Other outcomes

Two further prespecified outcomes showed no significant between-group difference: acquisition of multidrug-resistant organisms (four trials; Peto odds ratio 0.87, 95% CI 0.23 to 3.34) and hospital readmission (four trials; risk ratio 1.35, 95% CI 0.76 to 2.40), the latter dominated by a single trial (approximately 72% of the weight) and imprecise. Study-level data for both are given in Supplementary Table S1.

### Certainty of evidence

Certainty was assessed with GRADE for each pooled outcome (Table 2). For the co-primary consumption outcome (days of antibiotic therapy), certainty was low, downgraded for inconsistency (high I^2^, explained but not eliminated), imprecision in the adult subgroup, and study limitations (open-label trials and median-to-mean conversions). For the safety outcomes, all-cause mortality, bloodstream infection, treatment failure, fever recurrence, and invasive fungal infection, certainty was limited principally by imprecision arising from sparse events and by the high risk of bias of several contributing trials; mortality, supported by a precise absolute estimate, was the most secure of these. Per-outcome certainty ratings, with the reasons for downgrading and the anticipated absolute effects, are summarized in Table 2.

**Table 2:** Summary of findings: early discontinuation vs. continuation of empirical antibiotics in febrile neutropenia (GRADE).

Outcome	Participants (trials)	Relative effect (95% CI)	Risk with continuation	Absolute effect with early discontinuation (95% CI)	Certainty (GRADE)	Reason for the certainty rating
Co-primary outcomes						
Days of antibiotic therapy (total exposure)	941 (6)	—	Mean 5.1–14.4 days	MD − 3.43 days (−5.59 to −1.27); children −5.04, adults −1.52	⊕ ⊕ ○○ Low	Downgraded for risk of bias (open-label trials; means reconstructed from medians in 2 trials), inconsistency (I^2^ = 87%, largely explained by age), and imprecision in the adult subgroup, which did not further lower the overall rating.
All-cause mortality	1,007 (8); 1,679 (15) for absolute risk	Peto OR 1.23 (0.64 to 2.36)	35 per 1,000	2 more per 1,000 (10 fewer to 13 more)	⊕ ⊕ ⊕○ Moderate	Downgraded for imprecision (few events; optimal information size not met). The absolute estimate is precise and dominated by lower-risk-of-bias trials.
Secondary safety outcomes						
New bloodstream infection (afebrile at randomization)	716 (3)	RR 0.97 (0.34 to 2.77)	88 per 1,000	3 fewer per 1,000 (58 fewer to 156 more)	⊕ ⊕ ○○ Low	Downgraded for imprecision (wide confidence interval; few events) and risk of bias.
Treatment failure	1,296 (12)	RR 1.13 (0.80 to 1.61)	128 per 1,000	17 more per 1,000 (26 fewer to 78 more)	⊕ ⊕ ○○ Low	Downgraded for imprecision (confidence interval compatible with both benefit and harm) and risk of bias.
Fever recurrence	1,335 (9)	RR 1.08 (0.88 to 1.33)	179 per 1,000	14 more per 1,000 (21 fewer to 59 more)	⊕ ⊕ ○○ Low	Downgraded for risk of bias and imprecision (sparse events in several contributing trials).
Invasive fungal infection	619 (6)	Peto OR 1.56 (0.42 to 5.83)	73 per 1,000	36 more per 1,000 (41 fewer to 240 more)	⊕○○○ Very low	Downgraded for inconsistency (I^2^ = 68%, one small outlier trial), imprecision (very wide confidence interval), and risk of bias.

GRADE certainty: ⊕ ⊕ ⊕ ⊕ High; ⊕ ⊕ ⊕○ Moderate; ⊕ ⊕ ○○ Low; ⊕○○○ Very low.

Absolute effects: Risk with continuation is the pooled control-group event rate; the absolute effect with early discontinuation is calculated from the relative effect, except for mortality (pooled risk difference across all 15 trials). MD, mean difference; OR, odds ratio; RR, risk ratio; CI, confidence interval. Risk of bias was assessed independently by two reviewers.

In an exploratory analysis requested during review, trials were grouped by baseline risk (high-risk, 9 trials; low-risk, 4 trials; Supplementary Table S9). The reduction in antibiotic consumption was evident in both strata (high-risk mean difference −3.40 days, 95% CI − 5.70 to −1.10; the single low-risk trial reporting this outcome, ProFenC 2024, −3.69 days, −5.00 to −2.38). All-cause mortality did not differ in high-risk trials (Peto odds ratio 1.15, 95% CI 0.66 to 1.99), and no deaths occurred in either arm of the low-risk trials (absolute risk difference zero). Treatment failure (high-risk risk ratio 1.20, 95% CI 0.90 to 1.61; low-risk 0.75, 0.31 to 1.80) and fever recurrence (high-risk 1.07, 0.87 to 1.32; low-risk 0.60, 0.23 to 1.59) did not differ significantly between strata. Because baseline risk was confounded with trial era, disease mix, comparator duration, and stopping protocol, and the low-risk strata were small and contributed no deaths, this comparison is exploratory and hypothesis-generating. For the same reason, and because the biomarker-guided (ProFenC 2024) and early-de-escalation Ram et al. (2023) strategies were each represented by a single trial (Supplementary Table S3), outcomes could not be compared between individual discontinuation strategies, and the pooled estimate should be read as the effect of early discontinuation as a class rather than of any specific strategy.

## Discussion

In this systematic review and meta-analysis of 15 randomized controlled trials enrolling 1,692 participants with cancer and febrile neutropenia, early or short-course discontinuation of empirical antibacterial therapy substantially reduced antibiotic consumption without a detectable increase in mortality or major infectious complications. Days of antibiotic therapy fell by a mean of 3.43 days overall (95% CI − 5.59 to −1.27), an effect that was large and statistically robust in children (−5.04 days) but smaller and non-significant in adults (−1.52 days; test for subgroup differences *p* = 0.0002), and the direction was confirmed when consumption was re-expressed as antibiotic-free days within 28 days (+3.69 days). Across every safety outcome, the two strategies were statistically indistinguishable: all-cause mortality (Peto odds ratio 1.23; absolute risk difference 0.00, 95% CI − 0.01 to 0.01), new bloodstream infection (risk ratio 0.97), treatment failure (1.13), and fever recurrence (1.08). The outcomes most directly related to resistance and fungal superinfection—multidrug-resistant organism (MDRO) acquisition (0.87) and invasive fungal infection (1.56) were too sparse and heterogeneous to support firm conclusions, and should be regarded as inconclusive rather than reassuring.

The central message of these data is one of antimicrobial stewardship. Days of therapy is the metric by which stewardship programs increasingly track and benchmark antibacterial use, and a reproducible reduction of 3 to 5 days per febrile episode accrued across the many episodes a neutropenic patient experiences represents a meaningful contraction of broad-spectrum exposure in precisely the population in which exposure is heaviest. This matters because cumulative carbapenem and other broad-spectrum use is a principal driver of resistance among Gram-negative bloodstream pathogens in hematology and transplant units, where multidrug-resistant infection is now common and carries substantially higher mortality when empirical cover is inadequate (Baccelli et al., 2025; Park et al., 2024).

The safety findings are reassuring but require careful reading. Mortality was the most secure of these outcomes: although the relative estimate was imprecise, the absolute risk difference across all 15 trials was centered on zero with an interval of approximately ±1 percentage point, which reduces but does not exclude a clinically relevant difference: an absolute increase approaching one percentage point could still matter in otherwise low-risk patients, deaths were captured over differing follow-up windows, and individual trials were underpowered for death. For bloodstream infection, restricting the primary analysis to the contemporary scenario patients afebrile at randomization in whom new, post-randomization bacteremia was counted showed no difference, whereas the apparent upward shift seen only when older trials that stopped antibiotics during persistent fever were added (1.59, 95% CI 0.55 to 4.63) is best explained by unmasking of occult infection under that now-obsolete strategy rather than by a hazard of stopping in stable, afebrile patients. Treatment failure and fever recurrence likewise showed no signal, with a particularly narrow interval for recurrent fever. Together, these results support discontinuation in clinically stable, afebrile patients without a documented source, while leaving appropriate caution for high-risk adults and those with persistent fever. The trials differed materially in the population enrolled fever of unknown origin, clinically or microbiologically documented infection, persistent fever, or virus-positive episodes and in the stopping criteria applied (Supplementary Tables S2–S6); the primary conclusion therefore applies to clinically stable, afebrile patients with fever of unknown origin, whereas the historical persistent-fever and virus-positive trials address different clinical questions and the evidence in high-risk adults and cellular-therapy recipients remains limited.

At the same time, our review exposes a critical evidence gap that is central to this field: the very outcomes through which reduced antibiotic exposure should translate into patient benefit- MDRO acquisition, *Clostridioides difficile* infection, and invasive fungal disease are the least well studied. Only four trials reported MDRO acquisition and six reported invasive fungal infection, the latter with substantial heterogeneity (I^2^ = 67.9%) driven by a single small, high-risk trial, so neither benefit nor harm could be established. In sensitivity analyses that re-pooled every dichotomous outcome using Mantel–Haenszel methods without continuity correction, the estimates were concordant with the primary analyses, and the higher Peto estimate for invasive fungal infection proved to reflect the behavior of that method in the presence of a zero-event historical trial rather than a true excess (Mantel–Haenszel odds ratio 1.04, 95% CI 0.55 to 1.95). When the historical trials that discontinued antibiotics during persistent fever (Björnsson et al., 1977; Pizzo et al., 1979; Pizzo et al., 1982) were separated from contemporary trials, the only apparent safety signals in treatment failure and mortality were confined to the historical group (treatment failure: contemporary risk ratio 0.97, 95% CI 0.72 to 1.30, vs. historical 3.09, 1.49 to 6.41), whereas contemporary trials that stopped only after defervescence showed no increase in any safety outcome (Supplementary Table S7). The mechanistic expectation that shorter therapy lowers resistance and fungal superinfection, therefore, remains, for this population, largely untested in randomized data. Future trials in immunocompromised hosts should be designed and powered to capture these resistance- and stewardship-relevant endpoints directly, alongside microbiome and *C. difficile* outcomes, so that the downstream ecological benefits of early discontinuation can be measured rather than inferred.

Our findings are consistent with and extend earlier studies. The 2019 Cochrane review concluded that the evidence was low-certainty and rested on small, mostly older trials (Stern et al., 2019), and recent syntheses focused on adult hematology or early de-escalation reached broadly neutral safety conclusions (Ishikawa et al., 2023; Chen et al., 2025). By incorporating the newest randomized evidence including the trial by de Jonge and colleagues in adults with high-risk fever of unknown origin (de Jonge et al., 2022), a trial in recipients of hematopoietic stem-cell and CAR T-cell therapy (Ram et al., 2023), and recent pediatric trials in both low-risk (Kumar et al., 2021; Srinivasan et al., 2024) and high-risk (Chellapuram et al., 2025; Vissing et al., 2026) disease and by stratifying by age and grading certainty with GRADE, the present review provides a more current and granular synthesis. The pattern also aligns with the direction of guideline change: the 2023 pediatric fever-and-neutropenia guideline now permits discontinuation in well, afebrile children with negative cultures (Lehrnbecher et al., 2023), and the 2024 AGIHO adult guideline supports early de-escalation or discontinuation after defervescence irrespective of neutrophil count (Sandherr et al., 2025). Our data reinforce this stewardship-forward stance—most strongly in children while indicating that the adult evidence, though directionally favorable, remains less definitive.

Strengths of this review include its restriction to randomized trials, an up-to-date search capturing trials published through 2026, inclusion of stem-cell and CAR T-cell recipients, a prespecified age-stratified analysis, harmonization of the bloodstream-infection definition before pooling, and formal GRADE appraisal. Several limitations temper the conclusions. Heterogeneity in the consumption outcome was high, although it was largely explained by age and by the two trials whose means were reconstructed from medians. Restricting to directly reported means reduced I^2^ to 39% and left the estimate essentially unchanged. The consumption benefit was concentrated in pediatric trials, and the adult estimate did not reach significance, so adult and cellular-therapy evidence remains comparatively thin. Events were sparse for the safety, resistance and fungal outcomes, yielding wide confidence intervals and low-to-very-low certainty; five contributing trials were old and at high risk of bias; treatment-failure definitions varied; most contemporary trials were necessarily open-label; the search was limited to English-language reports; and several prespecified outcomes (sepsis, intensive-care admission, *C. difficile* infection, length of stay) and trial sequential analysis could not be undertaken because too few trials contributed data. Because age was confounded with trial era, risk category, disease mix, and comparator duration, the larger reduction seen in children should be read as an association rather than a pure age effect. The included trials also differ in what “early discontinuation” meant: the older trials stopped antibiotics during persistent fever, whereas contemporary trials stopped only after defervescence and clinical stability, so the pooled estimates combine strategies that are no longer considered equivalent. Two trials contributed febrile episodes rather than individual patients (Klaassen et al., 2000; Vissing et al., 2026), and treating episodes as independent may modestly narrow the confidence intervals for the affected outcomes. Days of therapy and antibiotic-free days within 28 days were reported differently across trials, further contributing to the observed heterogeneity. The 95% prediction interval for the consumption outcome crossed the null (−8.76 to 1.90 days), so a future trial could plausibly show no reduction.

For clinical practice, these results support early, protocolized discontinuation of empirical antibacterials as an option restricted to clinically stable, afebrile patients without a documented infection, particularly children and low-risk episodes, with individualized judgment retained for high-risk adults, persistent fever, or clinical instability. For research, adequately powered trials in adults and in cellular-therapy recipients are the priority, and they should report resistance and fungal endpoints (MDRO acquisition, *C. difficile* infection, invasive fungal infection), evaluate biomarker-guided stopping rules, and adopt standardized outcome definitions so that future syntheses can quantify both the clinical and the ecological effects of shorter therapy with greater certainty.

## Conclusion

In patients with cancer and febrile neutropenia, early discontinuation of empirical antibacterial therapy meaningfully reduces antibiotic consumption, a direct antimicrobial-stewardship benefit without a detectable increase in mortality or major infectious complications, with the clearest effect in children and in clinically stable, afebrile patients. The certainty of the safety evidence is limited by sparse events and older high-risk trials, and the resistance- and fungus-related outcomes most relevant to immunocompromised hosts remain insufficiently studied. In carefully selected, clinically stable, and afebrile patients without a documented bacterial infection, early discontinuation can reduce broad-spectrum exposure, with the clearest evidence in children and low-risk episodes, whereas clinically important harm remains insufficiently excluded in several high-risk adult and cellular-therapy settings, and multidrug-resistant organism acquisition and invasive fungal infection remain inconclusive rather than neutral. The reassurance on mortality rests on a precise absolute risk difference rather than on the relative estimate, which remains imprecise. Adequately powered adult and cellular therapy trials reporting resistance and fungal endpoints are needed to confirm safety and define the ecological benefits of shorter therapy.

## Data Availability

The original contributions presented in the study are included in the article/Supplementary material, further inquiries can be directed to the corresponding author.
