# Supplementary material for: Early discontinuation vs. continuation of empirical antibiotics in patients with cancer and febrile neutropenia: a systematic review and meta-analysis of randomized controlled trials
Source: Front Microbiol. 2026 Sep 25;17:1925996. doi: 10.3389/fmicb.2026.1925996 (PMC13650014; doi:10.3389/fmicb.2026.1925996)
Supplement: Supplementary file 1 [file Supplementary_file_1.DOCX]

**Supplementary Material**

*Early discontinuation versus continuation of empirical antibiotics in patients with cancer and febrile neutropenia: a systematic review and meta-analysis of randomised controlled trials*

**Supplementary Methods S1. Search strategy, as executed.**

Coverage: database inception to 1 June 2026; reports restricted to English language. The executed combination in MEDLINE and Embase was #1 AND #2 AND #4 (sensitivity-maximising). Concept #3 (antibiotic terms) is documented as a higher-precision alternative that was NOT applied in the executed search.

| **Source** | **Platform / provider** | **Date searched** | **Records** |
| --- | --- | --- | --- |
| **MEDLINE** | PubMed (U.S. National Library of Medicine) | 1 June 2026 | 1,671 |
| **Embase** | Embase.com (Elsevier) | 1 June 2026 | 3,499 |
| **Cochrane CENTRAL** | Cochrane Library (Wiley) | 1 June 2026 | 607 |
| **Trial register** | ClinicalTrials.gov | 1 June 2026 | 201 |
| **Total records** | — | — | 5,978 |

*After combination and de-duplication, 5,081 duplicate records were removed, 289 were removed by automation tools and 145 for other reasons (5,515 in total), leaving 463 records for title/abstract screening; the full flow is shown in the PRISMA 2020 diagram (Figure 1).*

**1. MEDLINE (PubMed) — executed combination #1 AND #2 AND #4**

```
#1 - FEBRILE NEUTROPENIA (POPULATION)
"Febrile Neutropenia"[Mesh]
OR ("Neutropenia"[Mesh] AND ("Fever"[Mesh] OR "Fever of Unknown Origin"[Mesh]))
OR ((neutropeni*[tiab] OR neutropaeni*[tiab] OR granulocytopeni*[tiab] OR granulocytopaeni*[tiab] OR agranulocytos*[tiab])
    AND (fever*[tiab] OR febrile[tiab] OR afebrile[tiab] OR pyrexia*[tiab] OR FUO[tiab]))
OR "neutropenic sepsis"[tiab]

#2 - DISCONTINUATION / DURATION (INTERVENTION)
"Duration of Therapy"[Mesh] OR "Drug Administration Schedule"[Mesh] OR "Withholding Treatment"[Mesh] OR "Deprescriptions"[Mesh]
OR discontinu*[tiab] OR cessation[tiab] OR ceasing[tiab] OR ceased[tiab] OR withdraw*[tiab] OR withhold*[tiab] OR withheld[tiab]
OR stopp*[tiab] OR stopping[tiab] OR "short course"[tiab] OR "short courses"[tiab] OR "short term"[tiab] OR shorten*[tiab]
OR duration[tiab] OR de-escalat*[tiab] OR deescalat*[tiab] OR streamlin*[tiab] OR optimis*[tiab] OR optimiz*[tiab]
OR "early termination"[tiab] OR "fixed duration"[tiab]

#3 - ANTIBIOTIC (higher-precision alternative; NOT applied in the executed search)
"Anti-Bacterial Agents"[Mesh] OR antibiotic*[tiab] OR antibacterial*[tiab] OR "anti-bacterial"[tiab] OR antimicrobial*[tiab]
OR "anti-microbial"[tiab] OR empiric*[tiab] OR "antibiotic therapy"[tiab] OR beta-lactam*[tiab] OR carbapenem*[tiab]
OR cephalosporin*[tiab] OR piperacillin[tiab] OR tazobactam[tiab] OR cefepime[tiab] OR meropenem[tiab]

#4 - RCT FILTER (Cochrane HSSS, sensitivity- and precision-maximising, PubMed format)
(randomized controlled trial[pt] OR controlled clinical trial[pt] OR randomized[tiab] OR randomised[tiab]
 OR placebo[tiab] OR "clinical trials as topic"[mesh:noexp] OR randomly[tiab] OR trial[ti])
NOT (animals[mh] NOT humans[mh])
```

**Paste-ready executed PubMed query (#1 AND #2 AND #4)**

(("Febrile Neutropenia"[Mesh] OR ("Neutropenia"[Mesh] AND ("Fever"[Mesh] OR "Fever of Unknown Origin"[Mesh])) OR

((neutropeni*[tiab] OR neutropaeni*[tiab] OR granulocytopeni*[tiab] OR granulocytopaeni*[tiab] OR agranulocytos*[tiab])

AND (fever*[tiab] OR febrile[tiab] OR afebrile[tiab] OR pyrexia*[tiab] OR FUO[tiab])) OR "neutropenic sepsis"[tiab]))

AND (("Duration of Therapy"[Mesh] OR "Drug Administration Schedule"[Mesh] OR "Withholding Treatment"[Mesh] OR

"Deprescriptions"[Mesh] OR discontinu*[tiab] OR cessation[tiab] OR ceasing[tiab] OR ceased[tiab] OR withdraw*[tiab] OR

withhold*[tiab] OR withheld[tiab] OR stopp*[tiab] OR stopping[tiab] OR "short course"[tiab] OR "short courses"[tiab] OR

"short term"[tiab] OR shorten*[tiab] OR duration[tiab] OR de-escalat*[tiab] OR deescalat*[tiab] OR streamlin*[tiab] OR

optimis*[tiab] OR optimiz*[tiab] OR "early termination"[tiab] OR "fixed duration"[tiab])) AND ((randomized controlled

trial[pt] OR controlled clinical trial[pt] OR randomized[tiab] OR randomised[tiab] OR placebo[tiab] OR "clinical trials

as topic"[mesh:noexp] OR randomly[tiab] OR trial[ti]) NOT (animals[mh] NOT humans[mh])))

**2. Embase (Embase.com / Elsevier) — executed combination #1 AND #2 AND #4**

```
#1 - FEBRILE NEUTROPENIA
'febrile neutropenia'/exp
OR ('neutropenia'/exp AND ('fever'/exp OR 'fever of unknown origin'/exp))
OR (((neutropeni* OR neutropaeni* OR granulocytopeni* OR granulocytopaeni* OR agranulocytos*)
     NEAR/3 (fever* OR febrile OR afebrile OR pyrexia* OR FUO)):ti,ab,kw)
OR 'neutropenic sepsis':ti,ab,kw

#2 - DISCONTINUATION / DURATION
'treatment duration'/exp OR 'drug withdrawal'/exp OR 'treatment withdrawal'/exp
OR (discontinu* OR cessation OR ceasing OR ceased OR withdraw* OR withhold* OR withheld OR stopp* OR stopping
    OR 'short course' OR 'short term' OR shorten* OR duration OR 'de-escalat*' OR deescalat* OR streamlin*
    OR optimis* OR optimiz* OR 'early termination' OR 'fixed duration'):ti,ab,kw

#3 - ANTIBIOTIC (higher-precision alternative; NOT applied in the executed search)
'antibiotic agent'/exp OR 'antibiotic therapy'/exp
OR (antibiotic* OR antibacterial* OR 'anti-bacterial' OR antimicrobial* OR empiric* OR beta-lactam* OR carbapenem*
    OR cephalosporin* OR piperacillin OR tazobactam OR cefepime OR meropenem):ti,ab,kw

#4 - RCT FILTER (Embase) - applied as written below
'randomized controlled trial'/exp OR 'controlled clinical trial'/exp OR 'randomization'/exp
OR 'double blind procedure'/exp OR 'single blind procedure'/exp
OR (random* OR placebo* OR (doubl* NEAR/1 blind*) OR (singl* NEAR/1 blind*)):ti,ab OR trial:ti
NOT ('animal'/exp NOT 'human'/exp)
```

**3. Cochrane CENTRAL (Cochrane Library, Wiley) — no RCT filter; source restricted to Trials (CENTRAL); result set #12**

```
#1  MeSH descriptor: [Febrile Neutropenia] explode all trees
#2  MeSH descriptor: [Neutropenia] explode all trees
#3  MeSH descriptor: [Fever] explode all trees
#4  (#2 AND #3)
#5  ((neutropeni* OR neutropaeni* OR granulocytopeni* OR granulocytopaeni* OR agranulocytos*)
     NEAR/3 (fever* OR febrile OR afebrile OR pyrexia* OR FUO)):ti,ab,kw
#6  "neutropenic sepsis":ti,ab,kw
#7  (#1 OR #4 OR #5 OR #6)
#8  MeSH descriptor: [Withholding Treatment] explode all trees
#9  MeSH descriptor: [Duration of Therapy] explode all trees
#10 (discontinu* OR cessation OR ceasing OR ceased OR withdraw* OR withhold* OR withheld OR stopp* OR stopping
     OR "short course" OR "short term" OR shorten* OR duration OR de-escalat* OR deescalat* OR streamlin*
     OR optimis* OR optimiz* OR "early termination" OR "fixed duration"):ti,ab,kw
#11 (#8 OR #9 OR #10)
#12 (#7 AND #11)
```

*The [Duration of Therapy] descriptor (#9) is retained here as built. Because the free-text term "duration" in #10 retrieves the same records within the same OR set, the result set (#12) is unaffected whether or not this descriptor was available on the Wiley platform at the time of searching.*

**4. ClinicalTrials.gov**

Searched directly (not through a bibliographic-database filter) to capture completed-but-unpublished, ongoing, and planned randomised trials. Completed records were cross-checked against the MEDLINE/Embase/CENTRAL results at de-duplication; ongoing and not-yet-recruiting records were retained to map the current evidence base for the Discussion. WHO ICTRP and ISRCTN were not searched.

```
SEARCH AS ENTERED IN THE SEARCH FIELDS
Condition or disease : febrile neutropenia
Other terms          : discontinuation OR duration OR de-escalation OR deescalation
                       OR stop OR stopping OR withdrawal OR withhold OR cessation
                       OR "short course" OR "fixed duration"
Study type           : Interventional
Recruitment status   : All

EQUIVALENT SINGLE EXPRESSION (ADVANCED / EXPERT SEARCH BOX OR API)
( AREA[ConditionSearch] "febrile neutropenia" )
AND ( discontinuation OR duration OR "de-escalation" OR deescalation OR stop OR stopping
      OR withdrawal OR withhold OR cessation OR "short course" OR "fixed duration" )
AND ( AREA[StudyType] INTERVENTIONAL )
```

*English-language restriction: applied for feasibility of full-text assessment and data extraction; its potential effect is acknowledged as a limitation in the main text.*

**Table S1. Study-level data used in each meta-analysis (early discontinuation vs continuation).**

| **Study** | **Early discontinuation** | **Continuation** | **Effect (95% CI)** |
| --- | --- | --- | --- |
| **Days of antibiotic therapy — mean difference (days)** | | | |
| How2017 | 11.90 (6.30); 78 | 14.40 (7.20); 79 | −2.50 (−4.62 to −0.38) |
| deJonge2022 | 6.67 (4.49); 144 | 7.67 (3.75); 137 | −1.00 (−1.97 to −0.03) |
| Ram2023 | 3.00 (3.40); 50 | 5.10 (5.70); 41 | −2.10 (−4.08 to −0.12) |
| Danish2026 | 8.10 (5.90); 45 | 13.50 (6.20); 43 | −5.40 (−7.93 to −2.87) |
| AIIMS2025 | 4.77 (7.49); 140 | 11.07 (5.84); 138 | −6.30 (−7.88 to −4.72) |
| ProFenC2024 | 3.44 (1.87); 23 | 7.13 (2.60); 23 | −3.69 (−5.00 to −2.38) |
| **All-cause mortality — Peto odds ratio** | | | |
| AIIMS2025 | 7 / 140 | 11 / 138 | 0.61 (0.24 to 1.59) |
| Bjornsson1977 | 3 / 6 | 2 / 11 | 4.23 (0.51 to 35.18) |
| deJonge2022 | 5 / 144 | 1 / 137 | 3.70 (0.74 to 18.60) |
| How2017 | 1 / 78 | 3 / 79 | 0.37 (0.05 to 2.64) |
| Pizzo1979 | 2 / 17 | 0 / 16 | 7.42 (0.44 to 124.12) |
| Pizzo1982 | 5 / 16 | 8 / 34 | 1.48 (0.39 to 5.66) |
| Ram2023 | 2 / 59 | 3 / 51 | 0.57 (0.09 to 3.39) |
| Rodriguez1973 | 3 / 41 | 2 / 40 | 1.48 (0.25 to 8.97) |
| **New bloodstream infection, afebrile at randomisation — risk ratio** | | | |
| How2017 | 9 / 78 | 14 / 79 | 0.65 (0.30 to 1.42) |
| deJonge2022 | 15 / 144 | 13 / 137 | 1.10 (0.54 to 2.22) |
| AIIMS2025 | 7 / 140 | 4 / 138 | 1.73 (0.52 to 5.76) |
| **Treatment failure — risk ratio** | | | |
| DALFEN2021 | 2 / 38 | 2 / 37 | 0.97 (0.14 to 6.56) |
| deJonge2022 | 28 / 144 | 21 / 137 | 1.27 (0.76 to 2.12) |
| How2017 | 11 / 78 | 14 / 79 | 0.80 (0.39 to 1.64) |
| Klaassen2000 | 2 / 36 | 5 / 37 | 0.41 (0.09 to 1.98) |
| Pizzo1979 | 7 / 17 | 1 / 16 | 6.59 (0.91 to 47.76) |
| Pizzo1982 | 9 / 16 | 8 / 34 | 2.39 (1.14 to 5.03) |
| ProFenC2024 | 2 / 23 | 1 / 23 | 2.00 (0.19 to 20.55) |
| Ram2023 | 9 / 59 | 12 / 51 | 0.65 (0.30 to 1.41) |
| Rodriguez1973 | 12 / 41 | 10 / 40 | 1.17 (0.57 to 2.40) |
| Santolaya1997 | 2 / 36 | 3 / 39 | 0.72 (0.13 to 4.08) |
| Santolaya2017 | 4 / 84 | 3 / 92 | 1.46 (0.34 to 6.33) |
| Torres2024 | 3 / 69 | 4 / 70 | 0.76 (0.18 to 3.27) |
| **Fever recurrence — risk ratio** | | | |
| AIIMS2025 | 73 / 140 | 65 / 138 | 1.11 (0.87 to 1.40) |
| DALFEN2021 | 2 / 38 | 2 / 37 | 0.97 (0.14 to 6.56) |
| deJonge2022 | 23 / 144 | 18 / 137 | 1.22 (0.69 to 2.15) |
| How2017 | 11 / 78 | 14 / 79 | 0.80 (0.39 to 1.64) |
| Klaassen2000 | 2 / 36 | 5 / 37 | 0.41 (0.09 to 1.98) |
| ProFenC2024 | 2 / 23 | 3 / 23 | 0.67 (0.12 to 3.62) |
| Ram2023 | 14 / 59 | 12 / 51 | 1.01 (0.51 to 1.98) |
| Santolaya2017 | 4 / 84 | 0 / 92 | 9.85 (0.54 to 180.28) |
| Torres2024 | 3 / 69 | 0 / 70 | 7.10 (0.37 to 134.94) |
| **Invasive fungal infection — Peto odds ratio** | | | |
| Bjornsson1977 | 4 / 6 | 0 / 11 | 32.69 (3.36 to 318.04) |
| deJonge2022 | 6 / 144 | 2 / 137 | 2.65 (0.65 to 10.80) |
| How2017 | 4 / 78 | 10 / 79 | 0.40 (0.13 to 1.19) |
| Pizzo1979 | 3 / 17 | 5 / 16 | 0.49 (0.10 to 2.34) |
| Pizzo1982 | 2 / 16 | 6 / 34 | 0.69 (0.14 to 3.42) |
| Rodriguez1973 | 1 / 41 | 0 / 40 | 7.21 (0.14 to 363.52) |
| **MDRO acquisition — Peto odds ratio** | | | |
| How2017 | 3 / 78 | 4 / 79 | 0.75 (0.17 to 3.41) |
| Pizzo1982 | 0 / 16 | 1 / 34 | 0.23 (0.00 to 15.35) |
| Rodriguez1973 | 10 / 41 | 4 / 40 | 2.70 (0.86 to 8.49) |
| Santolaya2017 | 0 / 84 | 2 / 92 | 0.15 (0.01 to 2.36) |
| **Hospital readmission — risk ratio** | | | |
| AIIMS2025 | 41 / 140 | 30 / 138 | 1.35 (0.90 to 2.03) |
| deJonge2022 | 17 / 144 | 10 / 137 | 1.62 (0.77 to 3.41) |
| Klaassen2000 | 2 / 36 | 5 / 37 | 0.41 (0.09 to 1.98) |
| ProFenC2024 | 2 / 23 | 0 / 23 | 5.00 (0.25 to 98.64) |

*Continuous outcome (days of antibiotic therapy): mean (standard deviation); N analysed. Dichotomous outcomes: events / N. Effect estimates are per-trial mean differences, risk ratios or Peto odds ratios with 95% confidence intervals, as used in the corresponding meta-analysis.*

**Table S2. Population, risk stratum, and febrile-neutropenia episode classification of the included trials.**

| **Trial** | **Age group** | **Underlying disease** | **Risk stratum** | **Episode type** | **Neutropenia definition** | **Fever definition** |
| --- | --- | --- | --- | --- | --- | --- |
| **Rodriguez 1973** | Adult/>=15 y | Cancer | High (FUO) | FUO | PMN<1000 | >=101F / >=38.3C (non-transfusion) |
| **Björnsson 1977** | Adult | Cancer | High (persistent FUO) | FUO (persistent fever) | granulocytes<500 | >=38C (non-transfusion) |
| **How 2017** | Adult | Haematological malignancy / HSCT | High (FUO) | FUO | ANC ≤0.5×10⁹/L expected ≥7 days (high-risk FN; no aetiological diagnosis) | ≥38.3°C once or ≥38.0°C ×1h [INFERRED — IDSA 2010; numeric threshold in protocol appendix, not main text] |
| **de Jonge 2022** | Adult | Haematological malignancy / HSCT | High (FUO) | FUO | ANC <0.5 x10^9/L | >38.5°C once, or >38.0°C on 2 measurements ≥2h apart |
| **Ram 2023** | Adult | Post-cellular therapy (HSCT / CAR-T) | High | FUO | ANC <500 cells/μL, or expected fall to <500 within 48h | ≥38.3°C once, or ≥38.0°C sustained >1h, with ANC <500/μL (or expected <500 in 48h) |
| **Santolaya 1997** | Paediatric | Mixed cancer | Low/Mixed (FUO) | FUO | ANC<500 | >=38.7C x2 |
| **Klaassen 2000** | Paediatric | Mixed (ALL predominant) | Low | FUO | ANC<500 | >38.5C once / >38 x2 |
| **Santolaya 2017** | Paediatric | Cancer + viral RTI | Mixed (documented viral) | Documented-viral | ANC<=500 | >=38.5C / >=38 x2 |
| **DALFEN 2021 (Kumar)** | Paediatric | Cancer / chemotherapy | Low | FUO | ANC <500 cells/mm³, or expected fall to <500 within 48h; recovery = ANC ≥500 | >38.3°C (101°F) once, or >38.0°C (100.4°F) sustained over 1h (axillary) |
| **ProFenC 2024 (Srinivasan)** | Paediatric | Cancer / chemotherapy | Low | FUO | Low-risk FN (IDSA 2010 / dept protocol); standard-arm recovery = ANC >500/mm³ | ≥38.3°C once or ≥38.0°C ×1h [INFERRED — IDSA 2010; definition in Suppl. Table 1, not main text] |
| **Torres 2024** | Paediatric | Cancer + viral RTI | Mixed (documented viral) | Documented-viral | ANC<=500 | >=38.5C / >=38 x2 |
| **AIIMS 2025 (Chellapuram)** | Paediatric (2-18 y) | Acute leukaemia (induction) | High | FUO | ANC <500/uL | >38.3°C (101°F) once, or >38.0°C (100.4°F) sustained >1h; ANC <500/μL (or expected <500 in 48h) |
| **Danish 2026 (Vissing)** | Paediatric | Cancer (ALL/AML etc.); intensive chemo | High | FUO | ANC <0.5 x10^9/L anticipated >=7 more days | Per protocol |
| **Pizzo 1979** | Mixed/Paediatric (1-33 y) | Leukaemia / solid tumour | High (persistent FUO) | FUO | PMN <500/mm3 | Fever >38C x3/24h or >38.5C once |
| **Pizzo 1982** | Mixed (2-30 y) | Cancer | High (persistent FUO) | FUO (persistent fever) | ANC<500 | >38C x3 / >=38.5 once |

*Episode type from the pre-specified subgroup map. FUO, fever of unknown origin (no documented infection); "persistent fever" denotes the historical stop-during-fever design; "documented-viral" denotes trials confined to virus-positive respiratory episodes. Proportions of clinically versus microbiologically documented infection at enrolment were not separately extracted. [INFERRED] flags a threshold not stated in the trial main text.*

**Table S3. Intervention strategy, stopping criteria, comparator, decision rule, empirical regimen, and co-interventions.**

| **Trial** | **Intervention (what was stopped; trigger)** | **Comparator** | **Decision rule** | **Comparator type** | **Empirical regimen** | **Co-interventions / antifungal** |
| --- | --- | --- | --- | --- | --- | --- |
| **Rodriguez 1973** | Stop all (day 4, FUO) | Continue 10 more days (total 14 d) | Clinical | Continue-to-recovery | Carindacillin+cephalothin | None noted |
| **Björnsson 1977** | Stop all (persistent fever, day 3) | Continue base regimen + add chloramphenicol/clindamycin (10 d) | Clinical | Continue + escalation | Carbenicillin+cephalothin+gentamicin | Comparator ESCALATED: base regimen + chloramphenicol/clindamycin (10 d) |
| **How 2017** | Stop all after ≥72 h afebrile/stable | Continue until ANC recovery | Clinical | Continue-to-recovery | Antipseudomonal β-lactam: cefepime/ceftazidime 2g tid, meropenem 1g tid, imipenem 0.5g qid, or pip-tazo 4g tid — monotherapy or + aminoglycoside (amikacin) / fluoroquinolone / glycopeptide (vancomycin/teicoplanin) | None noted |
| **de Jonge 2022** | Short carbapenem course; stop all after defervescence | Extended carbapenem until neutrophil recovery | Clinical | Extended-fixed-course | Carbapenem | None noted |
| **Ram 2023** | De-escalation THEN discontinuation (EDD) | Continue per standard until recovery | Clinical | Continue-to-recovery | Pip-tazo 4.5g IV q6h (or ceftazidime 2g IV q8h if penicillin allergy); ± vancomycin; amikacin if unstable; carbapenem if persistently unstable; levofloxacin if pulmonary infiltrate | None noted |
| **Santolaya 1997** | Stop all (day 3, FUO, stable) | Continue until FN resolution | Clinical | Continue-to-recovery | Antistaph penicillin + 3rd-gen ceph/aminoglycoside | None noted |
| **Klaassen 2000** | Stop all at discharge (placebo arm) | Oral cloxacillin+cefixime until ANC>500 or 14 d | Clinical | Continue-to-recovery (oral) | IV piperacillin+gentamicin pre-randomisation | Comparator = oral cloxacillin + cefixime (step-down) |
| **Santolaya 2017** | Withhold all at 48 h (virus+, stable) | Maintain until episode end (7 d) | Clinical | Continue-to-recovery | Ceftriaxone (low) / ceftazidime+amikacin (high) | None noted |
| **DALFEN 2021 (Kumar)** | Stop all after 24–48 h afebrile | Continuation as oral antibiotics until recovery | Clinical | Continue-to-recovery | IV cefoperazone-sulbactam ± amikacin; oral switch (Arm B) = amoxicillin-clavulanate + levofloxacin | Comparator = oral amoxicillin-clavulanate + levofloxacin (step-down) |
| **ProFenC 2024 (Srinivasan)** | Biomarker-guided (procalcitonin) stop | Continuation (standard) | Biomarker-guided | Continue-to-recovery | Cefoperazone-sulbactam (platelets >20k) or pip-tazo (platelets <20k) ± amikacin | None noted |
| **Torres 2024** | Withhold all at 48–72 h (virus+, stable) | Maintain until episode end | Clinical | Continue-to-recovery | Per Latin-American consensus | None noted |
| **AIIMS 2025 (Chellapuram)** | Stop all after ≥72 h afebrile, no documented infection | Continue until ANC >=500/uL | Clinical | Continue-to-recovery | Dual antibiotics, ≥1 antipseudomonal; first-tier cefoperazone-sulbactam, amikacin, or levofloxacin (alone/combined) | None noted |
| **Danish 2026 (Vissing)** | Stop all IV after 48 h defervescence | Continue until neutrophil recovery (max 10 days/episode) | Clinical | Continue-to-recovery | Per local institutional guideline (no fixed protocol regimen); antibiotic choice site-dependent — not specified in publication (Vissing 2026, Lancet Child Adolesc Health) or registry NCT04637464 | None noted |
| **Pizzo 1979** | Stop all (persistent fever) | Continue antibiotics | Clinical | Continue-to-recovery | Keflin + gentamicin + carbenicillin | None noted |
| **Pizzo 1982** | Stop all (persistent fever, day 7) | Continue +/- amphotericin B until afebrile + ANC>500 | Clinical | Continue-to-recovery | Keflin+gentamicin+carindacillin | Comparator ± amphotericin B (control = pooled ± antifungal arms, n=34) |

*Intervention arm = early discontinuation / short course. "De-escalation THEN discontinuation" (Ram 2023) and biomarker-guided stopping (ProFenC 2024) are not identical to stopping all empirical therapy. Prophylactic-antibiotic resumption after stopping was not reported for most trials; oral step-down comparators are flagged. Björnsson 1977 and Pizzo 1982 comparators carried co-interventions that differ in spectrum/antifungal exposure, not only duration.*

**Table S4. Randomised and analysed populations, unit of analysis, follow-up window, and reconciliation of denominators.**

| **Trial** | **Randomised (total; I/C)** | **Analysis population used** | **Unit** | **Mortality / main follow-up window** | **Reconciliation note (where analysed ≠ randomised)** |
| --- | --- | --- | --- | --- | --- |
| **Rodriguez 1973** | 81 (41/40) | All randomised episodes | Episodes | NR (fever + ANC recovery) | Two day-4 randomisation strata (afebrile 30/26; persistent fever 11/14) pooled; 41/40 used for every outcome. Mortality denominators previously shown as 12/10 were a transcription error, now corrected |
| **Björnsson 1977** | 17 (6/11) | As randomised (6/11) | Patients | 4 weeks | Analysed = randomised |
| **How 2017** | 157 (78/79) | As randomised (78/79) | Patients | 28 (±2) days from start of EAT | Analysed = randomised |
| **de Jonge 2022** | 292 (147/145) | ITT 281 (144/137) | Patients | To recovery; mortality/AEs to 30 d post-recovery | N in analyses = ITT 144/137 (11 excluded overall) |
| **Ram 2023** | 110 (59/51) | ITT 110 (59/51); PP FN cohort 50/41 | Patients | 30 d from FN onset | DOT analysed 50/41 = per-protocol treated-FN cohort (trial randomised PRE-FN); event outcomes use ITT 59/51 |
| **Santolaya 1997** | 75 (36/39) | As randomised (36/39) | Patients | until ANC>500 | Analysed = randomised |
| **Klaassen 2000** | 73 (36/37) | Episode-level 73 (36/37) | Episodes (54 children) | To ANC recovery | Unit = febrile EPISODES (73 in 54 children) → clustering caveat |
| **Santolaya 2017** | 176 (84/92) | As randomised (84/92) | Patients | until fever + ANC>500 | Analysed = randomised |
| **DALFEN 2021 (Kumar)** | 75 (38/37) | As randomised (38/37) | Patients | Until ANC ≥500 or fever recurrence, within ≤10 days | Analysed = randomised |
| **ProFenC 2024 (Srinivasan)** | 46 (23/23) | As randomised (23/23) | Patients | 14 days (primary/secondary endpoints); 28 days (all-cause mortality) | Analysed = randomised |
| **Torres 2024** | 139 (69/70) | As randomised (69/70) | Patients | until fever + ANC>=500 (>=7 d) | Analysed = randomised |
| **AIIMS 2025 (Chellapuram)** | 280 (140/138) | mITT 278 (140/138) | Patients | To day 35 induction or ANC ≥500 | 1 excluded per arm; analyses use mITT 140/138 |
| **Danish 2026 (Vissing)** | 88 (45/43) | Episode-level 88 (45/43) | Episodes (70 children) | 28 d | Unit = febrile EPISODES (88 in 70 children) → clustering caveat; counts from abstract (full text pending) |
| **Pizzo 1979** | 33 (17/16) | As randomised (17/16) | Patients | Until afebrile + ANC >500/mm³ (×2, 24h apart); plus 30-day off-study monitoring | Analysed = randomised |
| **Pizzo 1982** | 50 (16/34) | As randomised (16/34) | Patients | until fever + neutropenia resolution | Analysed = randomised |

*I/C, intervention/control. Per-outcome analysed denominators are in Table S1. Analysed numbers differ from randomised because not all participants contributed to every outcome (e.g., Rodriguez 1973 mortality is the documented-infection subset; Ram 2023 days-of-therapy is the per-protocol treated febrile-neutropenia cohort). Two trials contributed febrile episodes rather than individual patients (Klaassen 2000; Danish 2026), analysed with a clustering caveat. Mortality was assessed over trial-specific windows.*

**Table S5. Bloodstream-infection outcome: harmonised definition and per-trial classification.**

| **Trial** | **Population at randomisation** | **BSI definition fit** | **Analysis role** | **Events (I vs C)** | **Source / note** |
| --- | --- | --- | --- | --- | --- |
| **Rodriguez 1973** | afebrile_FUO(2°) | unverified_count_discrepancy | pending_source | (7)/(41) vs (1)/(40) | Bjornsson cites Rodriguez: randomised AFEBRILE subset 30 d/c vs 26 continue; 2 documented-infection deaths in stop arm. Conflicts with workbook 7/41 v |
| **Björnsson 1977** | febrile_FUO | occult_unmasking | sensitivity | 4/6 vs 0/11 | CORRECTED 3->4 (4 Klebsiella bacteraemia: Table II + 'four episodes of Klebsiella sepsis'); randomised FEBRILE pts; CONTROL arm escalated (+chloramphe |
| **How 2017** | afebrile_FUO | new_bacteraemia | primary | 9/78 vs 14/79 | bacteraemia during follow-up; fewer with discontinuation |
| **de Jonge 2022** | afebrile_FUO | new_bacteraemia | primary | 15/144 vs 13/137 | SHORT trial; documented infection excluded at entry; counts from grade 3-5 AE table |
| **Ram 2023** | index_episode | index_BSI | excluded | 9/59 vs 10/51 | MDI=BSI but = documented pathogen of INDEX episode (HCT/CAR-T); discontinuation pts had NO new documented infection; not comparable to breakthrough BS |
| **Santolaya 1997** | afebrile_low_risk | new_bacteraemia_unverified | candidate_unverified | 1/36 vs 2/39 | source verification pending |
| **Klaassen 2000** | afebrile_low_risk | new_bacteraemia_unverified | candidate_unverified | 1/36 vs 0/37 | modern low-risk paed; source verification pending |
| **Santolaya 2017** | afebrile_viral_FN | new_bacteraemia_unverified | candidate_unverified | 0/84 vs 2/92 | ESBL Klebsiella in control; source verification pending |
| **DALFEN 2021 (Kumar)** | afebrile_low_risk | no_events | no_contribution | 0/38 vs 0/37 | double-zero; no contribution to RR |
| **Torres 2024** | afebrile_viral_FN | no_events | no_contribution | 0/69 vs 0/70 | double-zero; no contribution to RR |
| **AIIMS 2025 (Chellapuram)** | afebrile_no_infection | new_bacteraemia | primary | 7/140 vs 4/138 | negative blood culture required at entry; 7/73 & 4/65 among recurrent-fever = complete arm counts; full-arm denominator correct |
| **Pizzo 1979** | afebrile_FUO | new_bacteraemia | sensitivity | 2/17 vs 0/16 | Group III F-G+ (afebrile); 2 E.coli septicaemia after stopping; high RoB (single-rater restart decisions) |
| **Pizzo 1982** | febrile_FUO | occult_unmasking | sensitivity | 3/16 vs 1/34 | randomised persistently-febrile (F+G+) pts; 3 bacteraemia (stop) vs 1 E.coli septicaemia (continue: G2+G3 pooled n=34, incl amphotericin arm); high Ro |

*Harmonised definition: new, microbiologically confirmed bloodstream infection arising AFTER randomisation, counted per patient, EXCLUDING the index infection at enrolment. Primary analysis = trials randomising afebrile/stable patients; sensitivity = trials randomising persistently febrile patients (occult-infection unmasking). Ram 2023 (index, not breakthrough) excluded; Rodriguez 1973 counts unverified and excluded pending source verification.*

**Table S6. Outcome definitions, effect measures, contributing trials, and harmonisation notes.**

| **Outcome** | **Definition used in this review** | **Effect measure** | **k** | **Events** | **Harmonisation / analysis note** |
| --- | --- | --- | --- | --- | --- |
| **days of antibiotic therapy DOT** | Total days of systemic antibacterial therapy (28-d frame where applicable); medians converted to means via Wan 2014 for de Jonge 2022 and AIIMS 2025; Danish 2026 episode-level | MD (days) | 6 | n/a | Headline figure. Pooled MD -3.4d (HKSJ -5.8 to -1.1), robust to leave-one-out. I2=87% -> lead with age strata: adult -1.5d (HKSJ crosses 0), |
| **antibiotic free days 28d** | 28 − days on systemic antibacterials within a fixed 28-day window from treatment initiation. Prophylactic agents NOT separately excluded | MD (days) | 2 | n/a | Within-28d consistency check (How2017 + Danish2026). MD +3.9d. Too few to pool formally; supports DOT direction. |
| **all cause mortality** | Death from any cause at the latest trial-reported follow-up (windows differ; see Table S4) | Peto OR (primary) or RD (shows all 15) | 8 (OR) / 15 (RD) | 58 | 7/15 are double-zero -> drop from OR/RR. Use Peto OR (forest=8) OR risk difference (forest=15, all studies). Do NOT funnel (zero-event heavy |
| **treatment failure** | Trial-specific composite: ranges from antibiotic restart for recurrent fever to a composite of documented infection, readmission, ICU transfer, persistent fever or death — HARMONISATION to a common definition PENDING | RR (or OR) | 12 | 175 | Cleanest large forest + funnel-eligible. HARMONISE the failure definition first (recurrence-restart vs composite) or repeats the AFD-28 prob |
| **bloodstream infection** | New microbiologically confirmed post-randomisation bacteraemia (harmonised; see Table S5) | RR (or OR) | 11 | 104 | Funnel-eligible numerically BUT definitionally fragile: AIIMS2025 (recurrent-fever-subset numerator), Rodriguez1973/Bjornsson1977 (count dis |
| **fever recurrence** | New fever after defervescence/discontinuation (trial-defined) | RR (or OR) | 9 | 253 | Well-populated (253 events). Just under funnel threshold. |
| **invasive fungal infection** | Proven/probable invasive fungal infection (trial-defined; criteria differ across five decades) | RR or Peto OR | 6 | 43 | Moderate; some sparsity -> consider Peto OR. |
| **mdro acquisition** | New isolation/colonisation with a multidrug-resistant organism (trial-defined) | RR or Peto OR | 4 | 24 | Small/sparse; interpret cautiously. |
| **hospital readmission** | Unplanned readmission (trial-defined window) | RR | 4 | 107 | Small but reasonable event count. |

*k = trials contributing to the pooled estimate (studies with events and a denominator in both arms; double-zero trials excluded from ratio measures). Treatment failure and fever recurrence retain trial-specific definitions. Bloodstream-infection and invasive-fungal diagnostics changed materially across 1973–2026 (indirectness).*

**Table S7. Sensitivity analyses: Mantel–Haenszel pooling (without continuity correction) and separation of historical persistent-fever trials.**

| **Outcome** | **Primary analysis** | **Mantel–Haenszel (all trials)** | **Mantel–Haenszel (contemporary)** | **Mantel–Haenszel (historical persistent-fever)** |
| --- | --- | --- | --- | --- |
| **All-cause mortality** | Peto OR 1.22 (0.63–2.34) | 1.13 (0.65–1.97); k=8 | 0.84 (0.43–1.63); k=5 | 2.44 (0.84–7.09); k=3 |
| **Treatment failure** | RR 1.13 (0.80–1.61) | 1.13 (0.86–1.48); k=12 | 0.97 (0.72–1.30); k=10 | 3.09 (1.49–6.41); k=2 |
| **Fever recurrence** | RR 1.08 (0.88–1.33) | 1.10 (0.90–1.34); k=9 | 1.10 (0.90–1.34); k=9 | — |
| **Invasive fungal infection** | Peto OR 1.56 (0.42–5.83) | 1.04 (0.55–1.95); k=6 | 0.90 (0.39–2.09); k=3 | 1.24 (0.48–3.18); k=3 |
| **MDRO acquisition** | Peto OR 0.87 (0.23–3.34) | 1.25 (0.53–2.92); k=4 | 1.34 (0.56–3.19); k=3 | — |
| **Hospital readmission** | RR 1.35 (0.76–2.40) | 1.35 (0.96–1.91); k=4 | 1.35 (0.96–1.91); k=4 | — |

*Risk ratios (RR) or odds ratios (OR) with 95% confidence intervals; k, contributing trials. Historical persistent-fever trials = Björnsson 1977, Pizzo 1979 and Pizzo 1982. Mantel–Haenszel estimates use no continuity correction; double-zero trials excluded; "—" = no contributing trials. Bloodstream infection is not repeated here (its contemporary/historical separation is the primary vs sensitivity analysis in the main text). The invasive-fungal Mantel–Haenszel estimate (1.04) is lower than the Peto estimate (1.56), indicating the higher Peto value reflected that method’s behaviour with the zero-event historical trial (Björnsson 1977).*

**Table S8. Outcome-specific risk-of-bias assessment (Cochrane RoB 2): domain-level judgements and supporting rationale.**

| **Study** | **D1** | **D2** | **D3** | **D4** | **D5** | **Overall** | **Support for judgement** |
| --- | --- | --- | --- | --- | --- | --- | --- |
| **All-cause mortality** | | | | | | | |
| **How 2017** | Low | Low | Low | Low | Low | **Low** | objective outcome, open-label unlikely to bias mortality; ITT; registered. |
| **de Jonge 2022** | Low | Low | Low | Low | Low | **Low** | objective; ITT; registered. (30d post-EON mortality used: 5/144 vs 1/137.) |
| **Ram 2023** | Some | Low | Low | Low | Some | **Some** | objective; single-centre + registration unclear (D1/D5 some concerns). |
| **AIIMS 2025 (Chellapuram)** | Low | Low | Low | Low | Low | **Low** | objective; mITT; registered; adequately powered. |
| **Danish 2026 (Vissing)** | Low | Low | Low | Low | Low | **Low** | 0 deaths both arms; registered; ITT. |
| **DALFEN 2021 (Kumar)** | Low | Low | Low | Low | Low | **Low** | no deaths; concealed allocation; registered. |
| **ProFenC 2024 (Srinivasan)** | Some | Low | Low | Low | Some | **Some** | no deaths in either arm; feasibility design. |
| **Klaassen 2000** | Low | Low | Low | Low | Low | **Low** | Double-blind, central pharmacy randomisation, ITT |
| **Santolaya 1997** | High | High | Low | High | High | **High** | Randomisation/concealment unclear; blinding questionable |
| **Santolaya 2017** | Some | Some | Low | Low | Some | **Some** | Open-label; allocation concealment unclear; blinded assessor; unregistered |
| **Torres 2024** | Some | Some | Low | Low | Some | **Some** | Open-label; allocation concealment unclear; blinded adjudicator; unregistered |
| **Rodriguez 1973** | High | High | Low | High | High | **High** | Old trial; randomisation unclear; open-label |
| **Björnsson 1977** | High | High | Low | High | High | **High** | Old trial; randomisation unclear; open-label; tiny |
| **Pizzo 1979** | High | High | Low | High | High | **High** | 1979 trial; randomisation/concealment unclear; open-label; tiny. |
| **Pizzo 1982** | High | High | Low | High | High | **High** | Old trial; randomisation unclear; open-label |
| **Antibiotic-free days (28 d)** | | | | | | | |
| **How 2017** | Low | Some | Low | Some | Low | **Some** | computer-generated allocation, masked pre-randomisation (D1 Low); open-label, stopping decision can be influenced (D2/D4 some concerns); ITT (D3 Low); registered NCT01581333, superiority (D5 Low). |
| **de Jonge 2022** | Low | Some | Low | Some | Low | **Some** | registered, ITT (D1/D3 Low); open-label, consumption protocol-driven (D2/D4 some concerns). |
| **Ram 2023** | Some | Some | Low | Some | Some | **Some** | single-centre, sealed-opaque-envelope concealment (D1 some concerns); open-label (D2/D4); ITT, 1 withdrawal (D3 Low); trial registration not identified (D5 some concerns). |
| **AIIMS 2025 (Chellapuram)** | Low | Some | Low | Some | Low | **Some** | computer randomisation, allocation concealment, data-analyst blinded, registered CTRI, adequately powered (D1/D5 Low); open-label (D2/D4); mITT, 1 excluded/arm with reasons (D3 Low). |
| **Danish 2026 (Vissing)** | Low | Some | Low | Some | Low | **Some** | computer block randomisation stratified by site, concealed (D1 Low); open-label, intervention defines exposure (D2/D4); ITT (D3 Low); registered NCT04637464, superiority (D5 Low). Unit=episode (clustering). |
| **DALFEN 2021 (Kumar)** | Low | Some | Low | Some | Low | **Some** | central concealed allocation, data-analyst blinded, registered NCT03003273 (D1/D5 Low); open-label (D2/D4); no loss to follow-up (D3 Low). NB AFD/DOT not yet extracted. |
| **ProFenC 2024 (Srinivasan)** | Some | Some | Low | Some | Some | **Some** | small feasibility RCT; randomisation/concealment & blinding unclear (D1/D2); 100% follow-up (D3 Low); 14-day window (note); registration to verify. |
| **Klaassen 2000** | Low | Low | Low | Low | Low | **Low** | Double-blind, central pharmacy randomisation, ITT |
| **Santolaya 1997** | High | High | Low | High | High | **High** | Randomisation/concealment unclear; blinding questionable |
| **Santolaya 2017** | Some | Some | Low | Low | Some | **Some** | Open-label; allocation concealment unclear; blinded assessor; unregistered |
| **Torres 2024** | Some | Some | Low | Low | Some | **Some** | Open-label; allocation concealment unclear; blinded adjudicator; unregistered |
| **Rodriguez 1973** | High | High | Low | High | High | **High** | Old trial; randomisation unclear; open-label |
| **Björnsson 1977** | High | High | Low | High | High | **High** | Old trial; randomisation unclear; open-label; tiny |
| **Pizzo 1979** | High | High | Low | High | High | **High** | 1979 trial; randomisation/concealment unclear; open-label; small randomised subset (33). |
| **Pizzo 1982** | High | High | Low | High | High | **High** | Old trial; randomisation unclear; open-label |
| **Bloodstream infection** | | | | | | | |
| **How 2017** | Low | Some | Low | Low | Low | **Low** | microbiologically defined bacteraemia (D4 Low); open-label (D2 some concerns); ITT; registered. |
| **de Jonge 2022** | Low | Some | Low | Some | Low | **Some** | bacteraemia ascertained via grade 3-5 AE reporting in open-label trial (D4 some concerns). |
| **Ram 2023** | Some | Some | Low | Some | Some | **Some** | MDIs (=BSI) are largely INDEX documented infections, not breakthrough -> closer to baseline balance than safety signal (definitional). |
| **AIIMS 2025 (Chellapuram)** | Low | Some | Low | Some | Low | **Some** | blood-culture growth among recurrent-fever subset (7/73 vs 4/65) entered against full-arm denominators; confirm no BSIs outside subset (D4 some concerns). |
| **Danish 2026 (Vissing)** | Low | Some | Low | Low | Some | **Some** | MDI excluded at entry -> BSI is incident/breakthrough (definitional); standalone count pending full text (D5 some concerns until reported). |
| **DALFEN 2021 (Kumar)** | Low | Some | Low | Some | Low | **Some** | low culture positivity (~1.2%) — confirm BSI count (0/0 entered). |
| **ProFenC 2024 (Srinivasan)** | — | — | — | — | — | **NA** | Outcome not reported by trial (BSI cells left blank). |
| **Klaassen 2000** | Low | Low | Low | Low | Low | **Low** | Double-blind, central pharmacy randomisation, ITT |
| **Santolaya 1997** | High | High | Low | High | High | **High** | Randomisation/concealment unclear; blinding questionable |
| **Santolaya 2017** | Some | Some | Low | Low | Some | **Some** | Open-label; allocation concealment unclear; blinded assessor; unregistered |
| **Torres 2024** | Some | Some | Low | Low | Some | **Some** | Open-label; allocation concealment unclear; blinded adjudicator; unregistered |
| **Rodriguez 1973** | High | High | Low | High | High | **High** | Old trial; randomisation unclear; open-label |
| **Björnsson 1977** | High | High | Low | High | High | **High** | Old trial; randomisation unclear; open-label; tiny |
| **Pizzo 1979** | High | High | Low | High | High | **High** | 1979 trial; randomisation/concealment unclear; open-label; counts Cochrane-coded. |
| **Pizzo 1982** | High | High | Low | High | High | **High** | Old trial; randomisation unclear; open-label |

*D1, randomisation process; D2, deviations from intended interventions; D3, missing outcome data; D4, measurement of the outcome; D5, selection of the reported result. "Some" = some concerns. Cell colour: green = low risk, yellow = some concerns, red = high risk, grey = not applicable.*

**Table S9. Risk-stratified estimates for the principal outcomes (high-risk versus low-risk trials).**

| **Outcome (measure)** | **High-risk trials** | **Low-risk trials** |
| --- | --- | --- |
| **Antibiotic consumption (days of therapy), mean difference** | -3.40 (-5.70 to -1.10); k=5 | -3.69 (single trial, ProFenC 2024) |
| **All-cause mortality, Peto odds ratio** | 1.15 (0.66 to 1.99); k=8 | No deaths in either arm (RD 0.00); OR not estimable |
| **Treatment failure, risk ratio** | 1.20 (0.90 to 1.61); k=6 | 0.75 (0.31 to 1.80); k=4 |
| **Fever recurrence, risk ratio** | 1.07 (0.87 to 1.32); k=4 | 0.60 (0.23 to 1.59); k=3 |

*Values are mean differences, risk ratios or Peto odds ratios with 95% confidence intervals; k, number of contributing trials. Trials were classified as high-risk (9 trials) or low-risk (4 trials) as shown in Supplementary Table S2; two virus-positive trials were not included in this contrast. Risk ratios use Mantel–Haenszel pooling without continuity correction. The comparison is exploratory and hypothesis-generating: baseline risk is confounded with trial era, disease mix, comparator duration and stopping protocol, the low-risk strata are small, and the low-risk trials contributed no deaths.*

**Figure S1. Sensitivity analyses for the co-primary consumption outcome, days of antibiotic therapy.**

**(A) Leave-one-out**

**(B) Directly reported means only (no median-to-mean conversion)**

**(C) Antibiotic-free days within a 28-day window**

*The pooled mean difference remained between −2.76 and −4.01 days on leave-one-out, was concordant when restricted to directly reported means (I² reduced to 39%), and was directionally consistent when re-expressed as antibiotic-free days within 28 days.*

**Figure S2.**  **RoB traffic-light**

**Figure S3. Contour-enhanced funnel plot for treatment failure.**

*Shaded bands mark conventional significance contours. Interpretation is cautious: only 12 clinically heterogeneous trials contributed, and funnel-plot assessment has limited power.*
